# Therapeutic ultrasound as a potential male contraceptive: power, frequency and temperature required to deplete rat testes of meiotic cells and epididymides of sperm determined using a commercially available system

**DOI:** 10.1186/1477-7827-10-7

**Published:** 2012-01-30

**Authors:** James K Tsuruta, Paul A Dayton, Caterina M Gallippi, Michael G O'Rand, Michael A Streicker, Ryan C Gessner, Thomas S Gregory, Erick JR Silva, Katherine G Hamil, Glenda J Moser, David C Sokal

**Affiliations:** 1 The Laboratories for Reproductive Biology, Department of Pediatrics, 220 Taylor Hall, CB7500, The University of North Carolina at Chapel Hill, Chapel Hill, North Carolina 27599, USA; 2 Department of Cell & Developmental Biology, CB7090, The University of North Carolina at Chapel Hill, Chapel Hill, North Carolina 27599, USA; 3 Department of Biomedical Engineering, 152 MacNider Hall, CB7575. School of Medicine, The University of North Carolina at Chapel Hill, Chapel Hill, North Carolina 27599, USA; 4 Integrated Laboratory Systems, 601 Keystone Park Drive, Suite 100, Durham, North Carolina 27713, USA; 5 FHI360, 2224 E. NC Highway 54, Durham, North Carolina 27713, USA; 6 Virginia Polytechnic Institute and State University, School of Biomedical Engineering and Sciences, Center for Injury Biomechanics, 440 ICTAS Building, Stanger Street, Mail Code 0194, Blacksburg, Virginia 24061, USA

**Keywords:** Male contraception, therapeutic ultrasound, testis, epididymis, wet-heat

## Abstract

**Background:**

Studies published in the 1970s by Mostafa S. Fahim and colleagues showed that a short treatment with ultrasound caused the depletion of germ cells and infertility. The goal of the current study was to determine if a commercially available therapeutic ultrasound generator and transducer could be used as the basis for a male contraceptive.

**Methods:**

Sprague-Dawley rats were anesthetized and their testes were treated with 1 MHz or 3 MHz ultrasound while varying power, duration and temperature of treatment.

**Results:**

We found that 3 MHz ultrasound delivered with 2.2 Watt per square cm power for fifteen minutes was necessary to deplete spermatocytes and spermatids from the testis and that this treatment significantly reduced epididymal sperm reserves. 3 MHz ultrasound treatment reduced total epididymal sperm count 10-fold lower than the wet-heat control and decreased motile sperm counts 1,000-fold lower than wet-heat alone. The current treatment regimen provided nominally more energy to the treatment chamber than Fahim's originally reported conditions of 1 MHz ultrasound delivered at 1 Watt per square cm for ten minutes. However, the true spatial average intensity, effective radiating area and power output of the transducers used by Fahim were not reported, making a direct comparison impossible. We found that germ cell depletion was most uniform and effective when we rotated the therapeutic transducer to mitigate non-uniformity of the beam field. The lowest sperm count was achieved when the coupling medium (3% saline) was held at 37 degrees C and two consecutive 15-minute treatments of 3 MHz ultrasound at 2.2 Watt per square cm were separated by 2 days.

**Conclusions:**

The non-invasive nature of ultrasound and its efficacy in reducing sperm count make therapeutic ultrasound a promising candidate for a male contraceptive. However, further studies must be conducted to confirm its efficacy in providing a contraceptive effect, to test the result of repeated use, to verify that the contraceptive effect is reversible and to demonstrate that there are no detrimental, long-term effects from using ultrasound as a method of male contraception.

## Background

An ideal male contraceptive would be inexpensive, reliable and reversible. Other desirable qualities include a low incidence of side effects, prolonged duration of the contraceptive effect and no need for invasive surgical procedures or hormonal treatments. Men have not had many options for non-invasive, side-effect-free, reliable contraception without resorting to the use of condoms. While the barrier method has proven to be a reliable method to prevent the spread of sexually transmitted diseases [1], it is not always accepted as a family planning method for committed, monogamous couples [1,2].

Ultrasound's potential as a male contraceptive was first reported by Fahim *et al. *[3]. In a series of publications, it was shown that a single application of ultrasound could result in a dramatic loss of germ cells from testes and that this loss of germ cells was reversible. No notable side effects other than infertility were reported during studies with rats, dogs and monkeys [4]. This method was tested on several human subjects who were already scheduled for orchiectomy to treat prostate cancer. These men reported that the procedure was pain-free, only creating a gentle feeling of warmth [4,5].

Fahim used frequencies, powers and a duty cycle associated with the therapeutic use of ultrasound rather than parameters used for imaging tissue. In addition, Fahim had an ultrasound generator and transducer built by Whitewater Electronics (Helenville, WI) specifically for use as a contraceptive device [4,5]. Unfortunately, this manufacturer is no longer in business and efforts to locate Fahim's original instrumentation have proved fruitless [personal communication, David Sokal, Family Health International].

Thus, the objective of this study was to determine if commercially available therapeutic ultrasound generators and transducers could replicate the loss of germ cells demonstrated by Fahim. We report that a present-day therapeutic ultrasound instrument was capable of inducing a nearly complete loss of germ cells from rat testes only when Fahim's original treatment conditions were modified.

## Methods

### Animals

All animal work was approved by the Institutional Animal Care and Use Committee (IACUC) of Integrated Laboratory Systems (ILS, Research Triangle Park, North Carolina, USA) or by the IACUC of the University of North Carolina (UNC, Chapel Hill, North Carolina, USA). Pilot Studies and Study 1 were performed at ILS while Study 2 was performed at UNC. Sprague Dawley rats (retired male breeders and adult females) were obtained from Charles Rivers Laboratories.

Male rats were anesthetized with isoflurane/oxygen (4% for induction, 2 - 2.5% to maintain anesthesia) prior to and during ultrasound treatment. A ligature was used to prevent retraction of the testes into the abdomen by the cremaster muscle during treatment.

### Ultrasound

A therapeutic ultrasound generator (ME740, Mettler Electronics, Anaheim, CA) and two different transducers (ME7413: 5 cm^2 ^surface area, 250 mm diameter; ME7410: 10 cm^2 ^surface area, 360 mm diameter; Mettler Electronics, Anaheim, CA) were used to treat rat testes. This instrument was capable of producing ultrasound of 1 or 3 MHz frequency with power up to a maximum of 2.2 W/cm^2 ^at a duty cycle of 100%. While the ME7413 transducer operated at both 1 MHz and 3 MHz, the larger ME7410 transducer only produced 1 MHz ultrasound.

### Treatment apparatus

**Figure 1:**
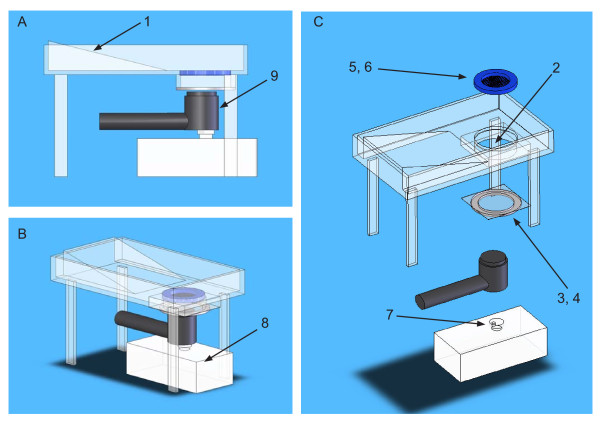
**Apparatus used to position rats for ultrasound treatment**. Parts were cut from Plexiglas unless otherwise noted. A slanted section supported most of the rat's body above the level reached by re-circulating coupling medium. The rat's scrotum was placed within the ultrasound treatment chamber after using a ligature to retain the testes within the scrotum (not shown). The bottom of the treatment chamber was formed of a single layer of latex, which was held in place against a rubber O-ring by an aluminum ring secured by machine screws. This formed a liquid-tight seal, allowing coupling medium to be re-circulated through the treatment chamber and a holding vessel contained within a temperature-regulated water bath (tubing, water bath, plumbing input and output have been omitted for clarity). A ring of ultrasound absorbing material was suspended 1 cm from the bottom of the treatment chamber to aid positioning of the testes and to reduce reflection of ultrasound energy. An ultrasound-transparent, nylon mesh was attached to the bottom of the ring to maintain a minimum distance of 1 cm between the bottom of the ultrasound chamber and the proximal portion of the scrotum.

A Plexiglas cylinder was used as the ultrasound chamber (70 mm diameter, 25 mm tall). The bottom of this chamber was a single layer of acoustically transparent latex. A single layer of acoustically transparent polypropylene mesh was held in place approximately 1 cm above the bottom of the chamber to provide a reproducible distance between the transducer and the scrotum. [Figure 1]

The ultrasound chamber was plumbed to allow input of coupling medium across the bottom of the chamber to dissipate any heat built up in the transducer. The transducer was affixed to an offset cam to allow it to rotate in a horizontal plane against the bottom of the ultrasound chamber during treatment. Ultrasound gel was used to coat the transducer face and the underside of the latex sheet used as the bottom of the ultrasound chamber to achieve acoustic coupling.

### Beam field mapping

The spatial distribution of acoustic pressures delivered by the ME7413 transducer to the testis was mapped as follows: a needle hydrophone (Onda, Sunnyvale, CA) was held vertically over the operating transducer and raster scanned 1.5 cm from the transducer's face (approximating the distance to the center of the testis) in 0.5 mm increments using a computer controlled motion stage (Newport, Irvine, CA). The beam field was mapped at 1 MHz and at 3 MHz with the transducer centered against the acoustically transparent latex sheet used as the bottom of the treatment chamber. Distilled water (DW), degassed distilled water and degassed 3% (w/v) sodium chloride were tested as coupling media. Both the ME7410 and ME7413 transducers were also mapped at 1 MHz frequency at distances of 0.5 cm to 3.5 cm from the transducer face.

### Determining the true effective radiating area (ERA) of our transducers

Beam plots acquired with the transducer - hydrophone separation set at 5 mm were used to determine the actual effective radiating area of both transducers used in our studies. Both transducers were driven at 1 MHz frequency and 1 W/cm^2 ^intensity with the Mettler Sonicator 740 used in our studies. The beam area was defined as the contiguous region with intensity greater than 5% of the peak value.

### Determining the true power output of our transducers

An Ultrasound Power Meter (model UPM-DT-1AV, Ohmic Instrument Co., Easton, MD) was used to measure the power output of our transducers at 1 or 3 MHz frequency, at intensities indicated by the Mettler Sonicator 740 to be 1 W/cm^2 ^and 2 W/cm^2^. The transducer face was centered 2 cm directly above the pressure-sensing cone and the radiant force method was used to determine the total output in Watts.

### Temperature data

An implantable copper-constantan thermocouple (IT-21, Physitemp Instruments, Clifton, NJ) was inserted down the long axis of the testis at an oblique angle to avoid piercing the epididymis to record testis temperature. The bimetal probe was connected to an analog-to-digital converter (Thermes USB, Physitemp Instruments, Clifton, NJ) and data was collected using Labview software (National Instruments, Austin, TX). Additional thermocouples were used to record the temperature of the coupling medium and the surface of the scrotum.

### Ultrasound treatment

The treatment frequency (1 MHz or 3 MHz), intensity setting (1 W/cm^2 ^to 2.2 W/cm^2^), duty cycle (100%) and duration were selected on the ultrasound generator [Tables 1, 2, and 3]. Rats were anesthetized and maintained on 2 - 2.5% isoflurane/oxygen. A ligature to retain the testes was tied tightly enough only to prevent the retraction of the testes from the scrotum during treatment. If testis temperature was recorded, the thermocouple was inserted at this time. The rat was positioned so that his scrotum was centered on the mesh layer of the ultrasound chamber. The appropriate coupling medium was circulated through the ultrasound chamber [Tables 1, 2, and 3]. The temperature of the coupling medium was controlled by re-circulating it through a holding vessel contained within a temperature-controlled bath. Temperature recording was initiated one minute prior to the start of ultrasound treatment and continued for one minute after the conclusion of ultrasound treatment to record pre- and post-treatment baseline temperatures.

**Table 1:** Treatment parameters for preliminary studies

Parameter	Preliminary Study #1	Preliminary Study #2
Coupling medium (**°**C)	N.R.	N.R.
Treatments	1	1
Duration (minutes)	10	10
Coupling medium	DW or PBS	dg-DW
Intensity (W/cm^2^)	1	2.2
Frequency (MHz)	1	1
Transducer (cm^2^)	5	5
Fertility Trial	No	Yes

The temperature of the coupling medium was not regulated (N.R.) in these studies. Coupling medium was distilled water (DW), phosphate buffered saline (PBS), or degassed, distilled water (dg-DW). Fertility trial was conducted as described in the Methods.

**Table 2:** Treatment parameters for Study 1

Group name	Sham	Wet heat	1 MHz, high power	3 MHz, high power	3 MHz, high power, Na+	1 MHz, low power	1 MHz, low power, Na+
Group number	1	2	3	4	5	6	7
Coupling medium (°C)	NR	45	37	37	37	NR	NR
Treatments	2	2	2	2	2	2	2
Duration (minutes)	15	15	15	15	15	15	15
Coupling medium	dg-DW	dg-DW	dg-DW	dg-DW	dg-Na^+^	dg-DW	dg-Na^+^
Intensity (W/cm^2^)	-	-	2.2	2.2	2.2	1	1
Frequency (MHz)	-	-	1	3	3	1	1
Transducer (cm^2^)	n/a	n/a	5	5	5	10	10
Rotation	n/a	n/a	+	+	+	-	-
Animals	2	3	3	4	4	3	3

Degassed, 3% sodium chloride was used as the coupling medium (dg-Na^+^), otherwise degassed distilled water was used (dg-DW). Temperature of the coupling medium was noted; otherwise there was no regulation (NR). Transducer ME7413 had a surface area of ~ 5 cm^2 ^(5) while model ME7410 had a surface area of ~ 10 cm^2 ^(10). Transducers were stationary (-) or were rotated in a plane parallel to the bottom of the ultrasound chamber (+). All groups received two consecutive treatments separated by two days.

**Table 3:** Treatment parameters for Study 2

Group name	Untreated	37C, 2 × 15, saline	37C, 2 × 10, saline	37C, 1 × 10	35C, 2 × 15, saline	35C, 2 × 15, water	35C, 2 × 10, saline, 2 W/cm^2^
Group number	8	9	10	11	12	13	14
Coupling medium (°C)	NR	37	37	37	35	35	35
Treatments	**-**	2	2	1	2	2	2
Duration (minutes)	**-**	15	10	10	15	15	10
Coupling medium	**-**	dg-Na^+^	dg-Na^+^	dg-Na^+^ or DW	dg-Na^+^	dg-DW	dg-Na^+^
Intensity (W/cm^2^)	**-**	2.2	2.2	2.2	2.2	2.2	2.0
Frequency (MHz)	**-**	3	3	3	3	3	3
Transducer (cm^2^)	**-**	5	5	5	5	5	5
Rotation	**-**	+	+	+	+	+	+
Animals	2	7	4	5	8	4	4

Degassed, 3% sodium chloride was used as the coupling medium (dg-Na^+^), otherwise distilled water (DW) or degassed, distilled water was used (dg-DW). Temperature of the coupling medium was noted, otherwise there was no regulation (NR). Transducer ME7413 had a surface area of ~ 5 cm^2 ^(5). Transducers were stationary (-) or were rotated in a plane parallel to the bottom of the ultrasound chamber (+). All groups received two consecutive treatments separated by two days, except as noted for Groups 8 and 11.

### Sperm count and motility were assessed two weeks after treatment

Preliminary Studies and Study 1: A testis and epididymis were removed prior to whole-body cardiac perfusion with Bouin's fixative. The cauda epididymis was carefully removed and several cuts were made to allow the release of sperm. The incised cauda epididymis was placed in 10 ml of M16 medium (Sigma, St. Louis, MO) for at least one half hour to allow motile sperm to be released. For determining sperm count, a dilution was made in distilled water and counted on a hemocytometer. Sperm count was expressed as millions of sperm per cauda epididymis. For estimating sperm motility, a dilution was made in M16 medium. Motile and non-motile sperm were scored visually using a hemocytometer.

Study 2: Sperm were collected from both cauda epididymides for determining sperm count, as described above. The total sperm count was determined using a hemocytometer by counting all sperm heads; the intact sperm count was calculated after tallying the number of sperm heads without an attached tail. Computer-aided sperm analysis performed with a CEROS sperm analysis system (software version 12.3; Hamilton Thorne Biosciences, Beverly, MA) was used to determine sperm motility.

### Sperm count index

Sperm counts (10^6 ^per cauda epididymis) were assigned to one of five arbitrary count ranges (< 11, 11-20, 21-40, 41-80, > 80). The count ranges were assigned values (from low to high) of: 0, 1, 2, 4 and 10. The Sperm Count Index was calculated as a weighted average using the arbitrary values assigned to the count ranges and the percentage of counts that fell within each range. For example, if 75% of a group's sperm counts fell in the second range of 11-20 × 10^6 ^and the remaining 25% of the counts fell in the fourth range of 41-80 × 10^6 ^the count index would be (0.75 × 1) + (0.25 × 4) = 1.75.

### Fertility testing

For each mating trial, a single male was housed with a pair of females for one week. In Pilot Study 2, the first mating trial was initiated the day of the ultrasound treatment. A second mating trial with a new pair of females occurred during the second week after ultrasound treatment. Sperm parameters were assessed at the conclusion of the second mating trial. Females were held for at least four weeks after the conclusion of their mating trial to complete pregnancies to term.

### Untreated, sham-treated or wet-heat controls

Three different controls were used for comparison of sperm counts and motilities. Untreated, retired breeders served as untreated controls. Sham-treated animals underwent all preparations for ultrasound treatment as treated animals: anesthesia was administered and maintained at 2 - 2.5% isoflurane/oxygen, scrotal fur was shaved, a ligature was used to retain the testes in the scrotum, room temperature coupling medium was placed in the treatment chamber, animal was placed on the treatment apparatus and the scrotum was centered in the treatment chamber. The temperature of the coupling medium was not regulated, the coupling medium was not re-circulated and the ultrasound generator was not turned on for the sham-treated animals. The wet-heat control animals were treated like the sham-treated controls except that the temperature of the coupling medium was held constant at 45°C while it was re-circulated through the treatment chamber.

### Histology

Pilot Studies and Study 1: Rats were anesthetized with isoflurane prior to cardiac perfusion with Bouin's fixative. One testis and one epididymis per animal were fixed for histological examination. An additional 24 hours of immersion fixation in Bouin's solution was performed prior to 2 days of washing in 70% ethanol. Tissues were processed into paraffin and 8 μm sections were stained with hematoxylin and eosin using standard methods. Digital micrographs were assembled into larger montages using the photomerge function in Photoshop CS (Adobe, San Jose, CA).

Study 2: Testes and epididymides were drop-fixed in Bouin's fixative for 24 hours to prepare them for histology. After an initial fixation of three hours, testes were cut into 0.5 cm thick cross-sections to facilitate penetration of Bouin's fixative. Fixed tissues were processed for histology as described above for Study 1. Digital micrographs were assembled into larger montages using an Olympus BX51 microscope and motorized 2-dimensional stage controlled by MetaMorph software (Molecular Devices, Sunnyvale, CA).

### Statistical analyses

One-way ANOVA analyses with post-tests were performed using GraphPad Prism version 5.0 d, GraphPad Software, San Diego California USA [6]. If data failed Bartlett's test for equal variances, significance was evaluated using the Kruskal-Wallis test and Dunn's multiple comparison post-test. In Study 1, sham-treated animals (n = 2) were excluded from analysis but the remaining treatment groups (n = 3 or 4) were analyzed for statistical differences.

## Results

### Field mapping and measuring the true ERA and power output of our transducers

**Figure 2:**
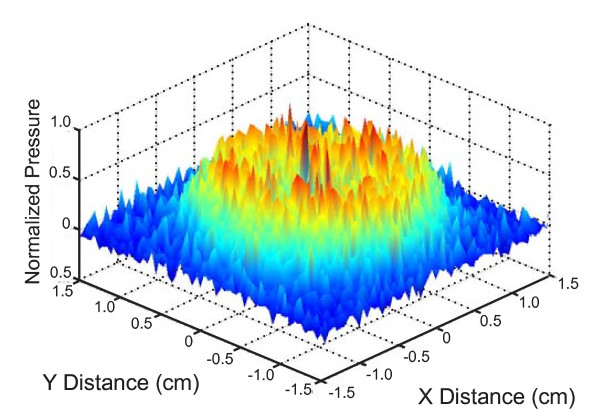
**Beam field map of the Model ME7413 therapeutic ultrasound transducer acquired at 3 MHz**. Normalized acoustic pressure is plotted on the Y-axis. The X and Y-axes represent the coordinates used to measure acoustic pressure delivered by the ultrasound transducer.

Field mapping of the output from the therapeutic transducer showed that there was a donut shaped "hotspot" in the 5-cm^2 ^transducer's output (ME7413) at 3 MHz [Figure 2]. The field map was the same regardless of the coupling medium used (DW, degassed DW or 3% (w/v) saline). The beam field of the 5-cm^2 ^transducer changed when it was mapped at 1 MHz: instead of a donut shaped hotspot, there was a discrete peak of energy near the center of the transducer face [Additional file 1 Figure S1].

Beam plots from both transducers [Additional file 1 Figure S1] were used to determine the area of the beam with energy equal to at least 5% of the peak beam energy when the distance between the hydrophone and transducer was set to 0.5 cm. The ME7413 transducer with a nominal area of 5 cm^2 ^had a true effective radiating area of 4.4 cm^2^; the ME7410 transducer with a nominal area of 10 cm^2 ^had a true effective radiating area of 9.3 cm^2^.

The power output of our transducers was determined at intensities indicated by the Mettler Sonicator 740 to be 1 W/cm^2 ^and 2 W/cm^2^. The 5 cm^2 ^transducer (ME7413) at a nominal intensity setting of 1 W/cm^2 ^had an output of 4.6 W at either 1 or 3 MHz; with a nominal intensity setting of 2 W/cm^2 ^the output varied from 8.9 Watts at 1 MHz to 9.3 Watts at 3 MHz. The 10-cm^2 ^transducer (ME7410) was only measured at 1 MHz and had an output of 10.2 Watts at a nominal intensity setting of 1 W/cm^2 ^and an output of 20.0 Watts at a nominal intensity setting of 2 W/cm^2^.

### True spatially averaged intensities were determined for our transducers

The 5-cm^2 ^transducer (ME7413) had an effective radiating area of 4.4 cm^2^. At both 1 and 3 MHz frequency the actual intensity for this transducer at an indicated 1 W/cm^2 ^was 1.05 W/cm^2^. The actual intensity for this transducer at an indicated 2 W/cm^2 ^varied from 2.02 W/cm^2 ^at 1 MHz to 2.11 W/cm^2 ^at 3 MHz. The spatially averaged intensities determined for this transducer were all within 6% of the values indicated by the Mettler Sonicator 740.

The 10-cm^2 ^transducer (ME7410) was only capable of operating at 1 MHz frequency and had an effective radiating area of 9.3 cm^2^. The actual intensity determined for this transducer at an indicated 1 W/cm^2 ^was 1.1 W/cm^2 ^and at an indicated 2 W/cm^2 ^the actual value was 2.15 W/cm^2^. The spatially averaged intensities determined for this transducer were within 10% of the values indicated by the Mettler Sonicator 740.

### Mitigating thermal bio-effects

In order to create a more even field of ultrasound at both frequencies, we devised a method to rotate the transducer in a horizontal plane coincident with the bottom surface of the ultrasound chamber with the center of rotation offset 8 mm from the center of the transducer face. The movement of the transducer mimics its use as a therapeutic device and results in an averaging of the field output over time.

The distance between the transducer and the scrotum was initially set to 3 cm. In an attempt to increase the energy delivered to the testes, the distance between the scrotum and the transducer was successively decreased. Some rats' testes actually rested on the bottom of the ultrasound chamber, separated from the transducer only by a layer of latex. This may have been responsible for some localized heat damage to the scrotum; these rats would occasionally develop a small circular discoloration on their scrotum.

Constructing a mesh support provided a reproducible offset of 1 cm between the bottom of the treatment chamber and the scrotum; recirculating the coupling medium eliminated any thermal bio-effects localized to the scrotum.

### Pilot study 1: published treatment parameters did not alter testis histology

Attempts to cause germ cell loss using a single ten minute dose of ultrasound at 100% duty cycle, 1 MHz and 1 W/cm^2 ^(Pilot Study 1) did not alter testis histology. These were the original parameters that were reported by Fahim to cause the loss of almost all germ cells from the testis [4]. Pilot study 1 used phosphate buffered saline or distilled water as the coupling medium filling the ultrasound chamber. The coupling medium surrounded the scrotum and allowed ultrasound to be efficiently transmitted from the transducer to the scrotum; ultrasound passed through the scrotum and was absorbed by the testes.

### Pilot study 2: increased power and degassed coupling medium

An experiment using a single treatment of 1 MHz at 2.2 W/cm^2 ^and 100% duty cycle through degassed water was performed (Pilot Study 2). Treating with 2.2 W/cm^2 ^was more successful than treating with 1 W/cm^2^. Two weeks after ultrasound treatment, the testis was depleted of developing germ cells and sperm count was reduced to 200 × 10^3 ^sperm per cauda epididymis. These sperm were not motile when analyzed in M16 medium.

Fahim reported that his ultrasound conditions caused rats to immediately lose their fertility [4]. When we treated with low frequency and high power (Pilot Study 2), pups were sired during the first and second weeks after treatment. However, there were no motile sperm at the end of this pair of one-week mating trials. Hypothetically, if another mating trial had been performed during the third week after treatment, the rat would have been infertile. This demonstrated that even though motile sperm were not detected at the end of the second mating trial, there were sufficient motile sperm during the initial two-week period after treatment for fertility.

### Study 1: two consecutive treatments

**Figure 3:**
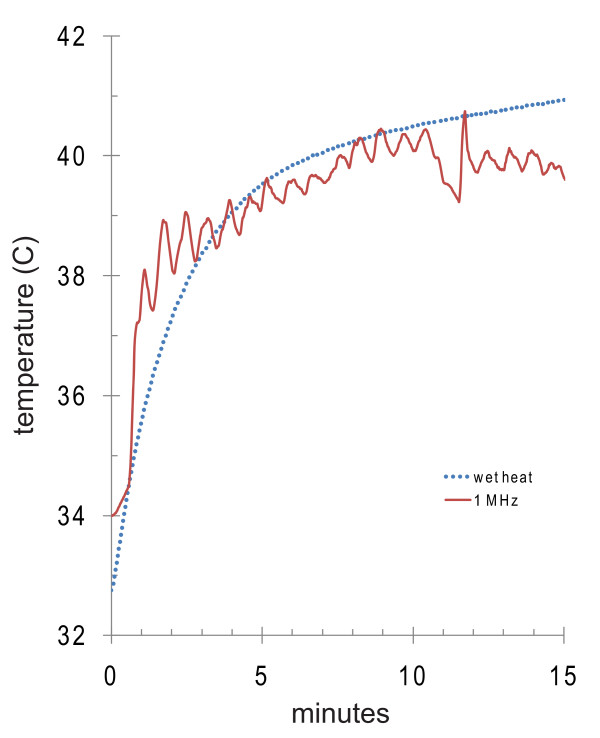
**Representative temperature curves during ultrasound or wet heat**. A thermal couple was inserted down the long axis of the testis and another was placed in the coupling medium. Coupling medium was re-circulated at 37°C during ultrasound treatments and at 45°C for the wet heat control. The rotation frequency of the transducer correlated with temperature fluctuations at the site of the thermal couple. The wet heat control yielded a testis temperature profile similar to an ultrasound treated testis.

In an attempt to bring post-treatment sperm counts closer to zero, the effect of two consecutive treatments separated by two days were tested [Study 1, Table 2]. Two weeks after treatment, total sperm count in the cauda epididymis dropped below 2 × 10^6 ^total sperm with essentially no motility when 3 MHz ultrasound was applied at 2.2 W/cm^2 ^through 37°C distilled water at 100% duty cycle [Table 4 Group 4]. Using coupling medium heated to 45°C allowed us to achieve internal testis temperatures comparable to the ultrasound treated testes [Figure 3]. Interestingly, heat alone [Table 4 Group 2] was more effective at reducing epididymal sperm count than the use of 1 MHz ultrasound either when the temperature of the coupling medium was held constant at 37°C [Table 4 Group 3, Tukey's post-test, p < 0.001] or when the temperature of the coupling medium was not regulated [Table 4 Group 6, Tukey's post-test, p < 0.001], however when 1 MHz ultrasound was applied through 3% saline at low power, sperm count was reduced sufficiently so that there was no significant difference from wet heat.

**Table 4:** Testis temperatures and sperm parameters from Study 1

Group	Treatment	n	Testis temperature (°C)	Sperm count (10^6^)	Motility (%)
1	Sham	2	30.1 ± 0.8	380 ± 33	45 ± 3
2	Wet heat	3	42.6 ± 0.1	23 ± 4	22 ± 5.8
3	Low freq., high power	3	40.5 ± 1.2	84 ± 3 **§**	54 ± 2
4	High freq., high power	3	41.8 ± 0.6	1.9 ± 0.9 **†**	0.3 ± 0.3
5	High freq., high power, Na+	4	nd	1.5 ± 0.8 **†**	nd
6	Low freq., low power	3	42.1 ± 2.8	96 ± 17 **§**	39 ± 2
7	Low freq., low power, Na^+^	3	35.4 ± 1.9	51 ± 5	40 ± 2

Sperm analyses were performed two weeks after ultrasound treatment. Average sperm count represents millions of sperm per cauda epididymis ± SEM. % Motility represents the percentage of recovered sperm with forward motility ± SEM. The average maximum testis temperature during treatment is listed in degrees Celsius ± SEM. Not determined (nd). **§**, statistically greater than the wet-heat control (Group 2) by Tukey's post-test (p < 0.001). **†**, statistically lower than 1 MHz, high power (Group 3) and 1 MHz, low power (Group 6) by Tukey's post-test (p < 0.001).

In contrast, the use of 3 MHz ultrasound resulted in a total epididymal sperm count ~10-fold lower than wet heat alone but with almost 1,000 times fewer motile sperm recovered from the epididymis: 3 MHz treated animals [Table 4 Group 4] had ~ 6 × 10^3 ^motile sperm per cauda epididymis while wet heat treated animals [Table 4 Group 2] had ~5 × 10^6 ^motile sperm per cauda epididymis (derived from data presented in Table 4; motile sperm = total sperm × % motile).

### Study 1: combining heat and ultrasound more effective than heat alone

**Figure 4:**
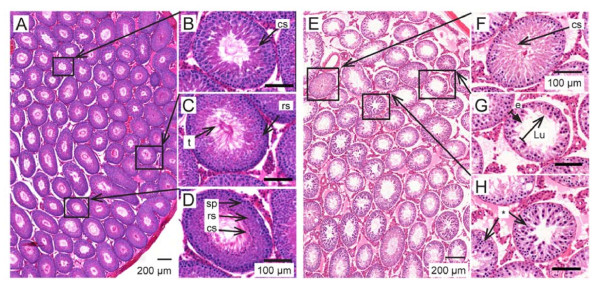
**Representative histology of normal or wet-heat-treated testes and seminiferous tubules**. A-D: hematoxylin and eosin stained cross-sections of untreated testis. The tall seminiferous epithelium contains many spermatocytes (sp), round spermatids (rs) and condensing spermatids (cs). Tails (t) of condensing spermatids and newly released testicular sperm are seen in the lumen (Lu) of some tubules. E-H: testis cross-section stained two weeks after wet heat treatment. Almost all tubules have enlarged luminal diameters after treatment with heat alone. The seminiferous epithelium (e) is reduced in height due to the loss of many spermatocytes and spermatids. Some tubules have disorganized epithelium (*).

The normal testis [Figure 4, A-D] had a complex epithelium consisting of many spermatogenic cells in various stages of spermatogenesis. Two weeks after using wet heat to elevate testis temperature there was a significant loss of spermatogenic cells although most seminiferous tubules still retained some spermatogenic cells [Figure 4, E-H].

**Figure 5:**
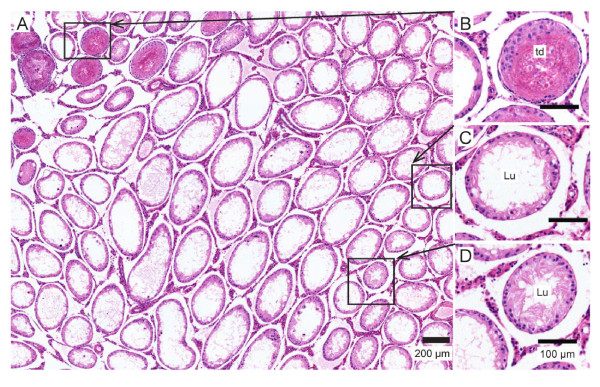
**Testis histology two weeks after 3 MHz ultrasound (Group 4)**. (A) The loss of spermatogenic cells after this treatment was more complete than after the wet heat treatment. This resulted in a shorter epithelium and a larger diameter lumen. (B) An isolated cluster of tubules in this particular animal showed evidence of thermal damage (td) in addition to the loss of spermatogenic cells. (C) Most tubules had a very short epithelial layer and increased lumen diameter due to the loss of all spermatocytes and spermatids. (D) Tubules that appear to have a larger epithelial layer and smaller diameter lumen were still missing spermatocytes and spermatids.

In contrast, combining elevated temperature and 3 MHz ultrasound [Table 4 Group 4 or 5] caused testis-wide depletion of germ cells [Figure 5]. The loss of developing spermatocytes and spermatids from the seminiferous epithelium was extensive; almost all tubules examined were effectively depleted by this treatment [Additional file 2 Figure S2]. The loss of spermatogenic output was reflected by sperm counts in these animals below 2 × 10^6 ^sperm per cauda epididymis, two weeks after ultrasound treatment [Table 4 Groups 4 and 5].

### Study 2: varying 3 MHz ultrasound treatments

**Figure 6:**
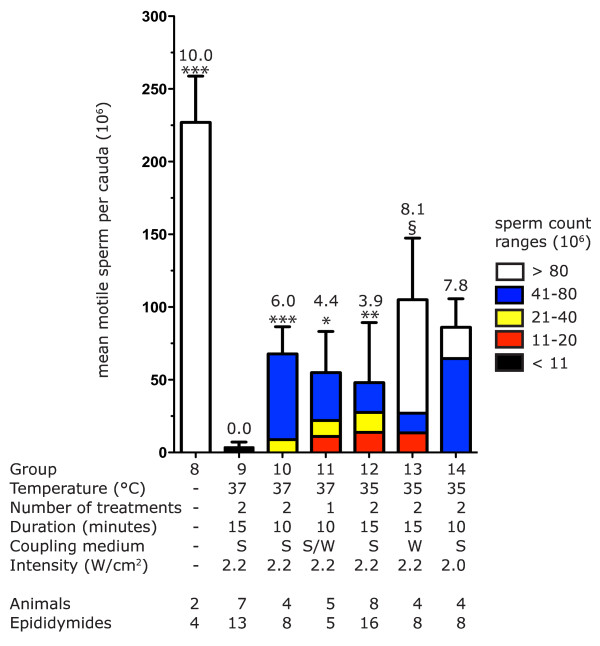
**Average and distribution of motile sperm counts from Study 2**. Motile sperm count was determined two weeks after treatment and was plotted as the mean ± SEM (10^6 ^per cauda epididymis). The stacked bars represent the proportion of sperm counts that fell into the following ranges of sperm counts (10^6 ^per cauda epididymis): < 11, 11 - 20, 21 - 40, 41 - 80, and > 80. Sperm Count Index was calculated as described in the Methods and is reported above each bar. Groups 8 - 12 failed Bartlett's test and were analyzed by the Kruskal-Wallis test with Dunn's post-test, symbols represent groups statistically different from Group 9: *, p < 0.05; **, p < 0.01; ***, p < 0.005. Groups 12 - 14 passed Bartlett's test: symbols represent groups statistically different from Group 12 by Tukey's post-test: **§**, p < 0.01.

All animals in Study 2 were treated with 3 MHz ultrasound. We varied the temperature of the coupling medium (35 or 37°C), its composition (DW or saline), the number (1 or 2) or duration of treatments (10 or 15 minutes) to determine the effect of these changes in treatment on mean motile sperm count per cauda epididymis [Figure 6]. Except for the group treated through degassed distilled water at 35°C (Group 13), all treatments resulted in a significantly lower mean motile sperm count than the untreated group (Group 8) according to Dunnett's multiple comparison test (p < 0.001).

The most effective treatment in Study 2 (Group 9: treating twice for 15 minutes at 3 MHz and 2.2 W/cm^2 ^intensity through degassed 3% saline held at 37°C) resulted in 3 ± 1 million motile sperm per cauda epididymis and a Sperm Count Index equal to 0. The next three lowest sperm counts were in Groups 10 - 12; all of these treatments resulted in mean motile sperm counts greater than 50 million sperm per cauda epididymis which was significantly higher than observed for Group 9 [Figure 6, Kruskal-Wallis with Dunn's post-test, refer to figure for p-values]. Group 12 had a Sperm Count Index equal to 3.9 and approximately one third of this group's sperm counts fell into the range of 41 - 80 million sperm per cauda epididymis. Group 10 had a Sperm Count Index of 6.0 with a mean sperm count of 67 ± 7 million motile sperm per cauda epididymis. As the higher Sperm Count Index indicated, a much larger proportion (7/8) of this group's sperm counts fell into the range of 41 - 80 million sperm per cauda epididymis.

### Study 2: saline was a more effective coupling medium than distilled water at 35°C

**Figure 7:**
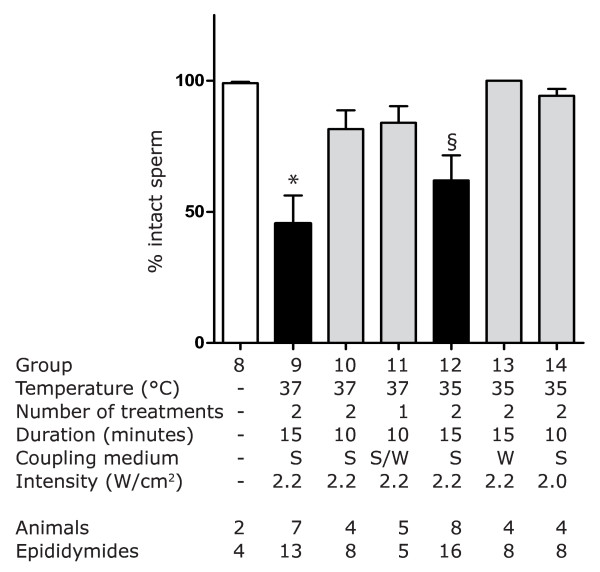
**Percentage of intact sperm recovered in Study 2**. Sperm counts tallied both intact sperm and sperm heads not attached to a tail. The number of intact sperm was expressed as a percentage of the total number of sperm recovered. *, Group 9 was statistically lower than Groups 8, 13 and 14 by Tukey's post-test (p < 0.01). **§**, Group 12 was statistically lower than Group 13 by Tukey's post-test (p < 0.05).

When animals were treated once at 37°C for 10 minutes at 2.2 W/cm^2 ^there was not a significant difference in sperm count as a function of coupling medium (degassed distilled water versus degassed 3% saline) so this data was pooled (Group 11). However, when animals were treated twice at 35°C for 15 minutes at 2.2 W/cm^2 ^the composition of the coupling medium did make a significant difference in sperm count (Tukey's post-test, p < 0.01): degassed 3% saline (Group 12) resulted in a sperm count 50% lower than degassed distilled water (Group 13). The use of saline resulted in about half of the sperm counts for Group 12 to be lower than 41 × 10^6 ^per cauda epididymis (Sperm Count Index = 3.9) while the use of distilled water (Group 13) resulted in only about 12% of counts below that threshold [Figure 6, Sperm Count Index = 8.1]. In addition, the number of intact sperm was significantly lower (Tukey's post-test, p < 0.05) when treating at 35°C through 3% saline [Figure 7, Group 12] than through degassed distilled water [Figure 7, Group 13].

### Most effective treatment

When the four treatments groups (Groups 9 - 12) with the lowest mean sperm counts in Study 2 were compared by one-way ANOVA, Group 9 was found to have a significantly lower mean motile sperm count than the other three groups (Kruskal-Wallis with Dunn's post-test, refer to Figure 6 for p-values). In addition, the percentage of intact sperm in Group 9 [Figure 7] was significantly lower (Tukey's post-test, p < 0.01) than the untreated control [Figure 7, Group 8]. Thus, the treatment that reduced cauda epididymis sperm count two weeks after treatment to the lowest levels was the same in Study 1 (Group 5) and in Study 2 (Group 9): two 15- minute treatments with 3 MHz ultrasound at 2.2 W/cm^2 ^through degassed 3% saline maintained at 37°C.

## Discussion

### Rat as a model system

Rats are reported to retain fertility even with extremely low sperm counts [7]. In contrast to rats, the World Health Organization has defined oligospermia in men as less than 20 million sperm/ml in the ejaculate and men are generally considered sub-fertile when their sperm concentration drops below 10 million sperm/ml [8]. Thus, we anticipate that decreasing sperm count sufficiently to cause infertility in rats would also cause infertility in men. However, sperm counts or concentrations that would represent infertility in men could allow rats to retain their fertility. Our second pilot study showed that the absence of motile sperm at the end of a mating trial did not rule out the ability to sire pups. With the mating scheme used in our study, it appeared that sperm count was changing rapidly and that the count on the day of conception could be higher than the count determined at necropsy. Consequently, in lieu of testing fertility we decided to assay epididymal sperm reserves to monitor the efficacy of our treatment conditions.

Our results clearly show that therapeutic ultrasound treatment depleted developing germ cells from the testis and subsequently decreased the size of sperm reserves in the epididymis when rats were treated with two consecutive ultrasound treatments separated by two days [Table 4 Figure 6]. This differs from reports in the 1970s by Fahim *et al. *[3,4], which reported that a single treatment of 1 MHz ultrasound was sufficient to induce a contraceptive effect of approximately six months duration. No mention of controlling the temperature of the coupling medium appeared in those original reports. In contrast, we found that combining elevated temperature, high power and high frequency was the most effective method for reducing sperm count.

### Variation between ultrasound transducers

A direct comparison between our treatments and those of Fahim are not possible without measuring the true effective radiating area (ERA, cm^2^) and power output (Watts) for all of the transducers used in these studies in order to calculate the true spatial average intensity (SAI, W/cm^2^) delivered during treatment. The SAI reported by clinical therapeutic ultrasound systems is not directly regulated in the United States by the Food and Drug Administration (FDA) even though this is the parameter most often used clinically to determine dosing during treatment. The FDA does require the true power output to be within ± 20% of the value reported by the manufacturer however no specific guideline was presented for the accuracy in reporting ERA [9]; most manufacturers report ERA with an error of ± 20 - 25%. Therefore, the true SAI for a transducer could vary by up to 150% from the displayed value while still satisfying FDA guidelines for ERA and power output. A study of sixty-six therapeutic ultrasound transducers showed that their true SAI varied from -43% to +63% of the displayed value [10]. The effects of ultrasound are dose-dependent, thus reproducible clinical dosing of therapeutic ultrasound requires determining the actual ERA, power output and SAI of the generator and transducers being used for treatment.

In some cases, more advanced monitoring techniques such as quantitative Schlieren assessment may be required to discern differences in output of transducers operated under identical nominal parameters [11]. This method can measure the power distribution in discrete portions of the ultrasound beam that are not captured by measurements mandated by the FDA such as beam non-uniformity ratio (BNR) and the aforementioned total power and ERA. Differences in the distribution of power within an ultrasound field may account for the ability of nominally identical transducers to heat tissue at significantly different rates [11].

We determined the actual effective radiating areas and power output of the transducers used in our studies. The true SAI of our transducers were determined to be within 10% of the values reported by our therapeutic ultrasound generator. In addition to determining the true ERA, power output and SAI for our transducers, we have also provided beam plots [Additional file 1 Figure S1] to facilitate comparison of our study results with future studies and to begin to standardize the clinical dosing of therapeutic ultrasound when used as a male contraceptive.

Since Fahim's custom-built generator and transducer were not available for testing, we cannot rule out the possibility that his system delivered more ultrasound energy to the testes than our therapeutic ultrasound instrument. Accordingly, we modified our coupling medium and treatment parameters to increase the delivery of ultrasound energy to the testes. While attempting to maximize energy delivery, we also took steps to mitigate any thermal bio-effects observed on the scrotal epithelium. The transducer face became quite hot to the touch by the end of each treatment so we reasoned that conductive transfer of heat caused occasional circular discolorations when the scrotum was pressed against the bottom of the treatment chamber. We modified the interior of our chamber to provide a reproducible offset between the scrotum and the chamber bottom/transducer. This also provided a space to re-circulate coupling medium between the scrotum and chamber bottom/transducer to dissipate any localized buildup of heat. Irregularities in the beam field prompted us to rotate the transducer to achieve a time averaging of the beam field energy. These modifications to the originally published protocol, especially the rotation of the transducer, may have caused a decrease in energy delivered to the testes. Rotating the beam field with an 8 mm offset cam caused a central area of continuous ultrasound exposure, surrounded by an area of lower, time-averaged ultrasound exposure. Time-averaging the beam field may account for the increased power, duration and number of treatments that we required to replicate Fahim's original result; the central area of continuous ultrasound exposure may account for the occasional thermal damage observed in some seminiferous tubules.

### Coupling medium

Our attempts to deplete germ cells using 1 MHz ultrasound at 1 W/cm^2 ^without controlling the temperature of the coupling medium were only partially successful [Study 1, Table 4 Group 6 and 7]. The use of 1 MHz ultrasound at either low or high power was less effective than the use of wet heat alone (p < 0.001) [Table 4; Group 2 versus Groups 3 or 6]. However, 1 MHz ultrasound decreased sperm count almost two-fold when the coupling medium was switched from degassed distilled water to 3% (w/v) sodium chloride in degassed distilled water [Table 4; Group 6 vs. Group 7]. The use of 3% sodium chloride and 1 MHz ultrasound [Table 4; Group 7] decreased sperm count to levels that were not statistically different from that achieved with wet heat alone [Table 4; Group 2].

When the temperature of the coupling medium was held at 37°C, 3 MHz ultrasound at 2.2 W/cm^2 ^decreased sperm count below 2 × 10^6 ^sperm per cauda in the presence or absence of saline [Study 1, Table 4 Group 4 and 5]. Attempting to reduce sperm count with the coupling medium held at 35°C was only partially successful [Study 2, Figure 6, Groups 12, 13, and 14]. However, the use of degassed 3% saline again caused a two-fold decrease in sperm count compared to the use of degassed distilled water [Figure 6, Group 12 vs. 13]; this drop in sperm count was statistically significant (p < 0.01). The number of intact sperm also decreased significantly (p < 0.05) when degassed 3% saline was used [Figure 7, Group 12 vs. 13]. When treatment conditions were less effective at reducing sperm count (combinations of degassed distilled water, lower temperature, lower power, or lower frequency) it appears that the addition of 3% saline to the coupling medium may cause a statistically significant drop in sperm count.

This corroborates a report in the literature that this coupling medium was more effective than distilled water alone when attempting to deplete germ cells from Macaque testes [4]. The biophysical basis for this phenomenon is currently unknown since the acoustic pressure delivered by our ultrasound transducers was not affected by either including 3% saline or by degassing our distilled water. However, 3% saline could have a biological effect on the scrotal epithelia or the dartos fascia (muscular tissue under the skin) that enhances the transmission of ultrasound energy. The dartos fascia is responsible for the furrowing of the scrotal skin, an adaptation related to thermal regulation of the testes. Although 3% saline failed to enhance the effect of 3 MHz ultrasound at 37°C, additional studies exploring different coupling media and the effect of their temperature are warranted.

## Conclusions

### Potential applications

Depleting spermatocytes and spermatids from testes non-invasively with therapeutic ultrasound has multiple applications. If the method proves to be reversible, it would provide a new tool for investigating spermatogonial expansion and differentiation. By creating testes depleted of differentiated spermatogenic or meiotic cells, investigators could test directly the effect of compounds proposed to regulate spermatogonia. In addition, spermatogonial stem cells are assayed by colony formation after transplantation into recipient testes depleted of germ cells by chemical treatment [12]. Rat spermatogonia can develop within the mouse seminiferous epithelium into spermatids that are morphologically distinct from those of the mouse [13,14]. Therefore, ultrasound-treated, syngeneic testes could serve as an alternatively prepared host for assaying spermatogonial stem cell numbers.

If the method can be made permanent, a non-invasive method for controlling various domestic pet populations could be developed. Leoci [15] has successfully used therapeutic ultrasound as a non-invasive method for canine sterilization. Fahim reported that his treatment method did not affect testosterone production by Leydig cells [4]. Thus, ultrasound treatment could be adopted as part of a larger strategy to control nuisance animal populations using the trap-neuter-return model [16,17]. Introducing sterile males into a population was effective in controlling insect populations [18] and was proposed to be effective in species where a dominant male breeds with a harem of females in a restricted territory such as white-tailed deer (*Odocoileus virginianus*) [19] or feral horses [20-22]. Controlling deer populations in urban or suburban areas would accrue many public health benefits since white-tailed deer carry ticks that transmit disease [23-25], are at risk for tuberculosis [26,27], and in the United States there are about 247 thousand collisions each year between deer and automobiles that damage approximately 1 billion dollars in property and kill approximately 200 people [28,29].

Ultimately, the most significant application of treating testes with ultrasound will be to address the global health issue of unintended pregnancies. The World Health Organization estimates that 228 million of the 600 million women of reproductive age worldwide are at risk for mistimed or unwanted pregnancy [30-32]. Yearly these unintended pregnancies result in almost 50 million abortions; almost half of these abortions are classed as unsafe, resulting in 47 thousand maternal deaths [33,34]. In the United States alone there are at least 3 million unintended pregnancies each year representing about 50% of all pregnancies [35,36]. Clearly, developing another safe, efficient and inexpensive method for contraception would only help to lower the rate of unwanted pregnancies and abortions. A permanent or reversible method of contraception based on therapeutic ultrasound treatment could encourage more men to share greater responsibility for family planning.

### Future studies

Future studies will determine if our ultrasound treatment parameters result in a reversible loss of fertility as previously reported by Fahim. The treatment that was most effective at reducing epididymal sperm count (3 MHz, 2.2 W/cm^2^, two 15-minute treatments separated by two days with coupling medium temperature maintained at 37°C) represents an upper limit for applying ultrasound to the testes since thermal bio-effects were noted in some treated tubules. Results from Study 2 showed that relatively small changes in treatment conditions caused statistically significant changes in sperm count when assessed two weeks after treatment. Longer-term studies will be required to determine if those treatment conditions cause a progressive loss of spermatogenic cells that ultimately results in the depletion of epididymal sperm reserves. A major goal of our future studies will be to determine the "minimum effective dose" of ultrasound that induces a reversible loss of fertility.

In conclusion, our results demonstrate that a short exposure to therapeutic ultrasound is an effective method for depleting testes of spermatogenic cells and reducing epididymal sperm reserves within two weeks of treatment. The odds of conceiving decrease linearly when sperm concentrations are below 40 million sperm/ml [37] and effective contraception occurs when hormonal treatment or vasectomy cause sperm concentration to fall below 3 million sperm/ml [38,39]. Our ability to use a widely available therapeutic ultrasound system to reduce motile sperm count below 5 million sperm per cauda epididymis just two weeks after treatment shows that therapeutic ultrasound holds great promise as the basis for a male contraceptive. Optimizing the treatment conditions, studying the safety of repeated use, the duration of the contraceptive effect and its reversibility and are the next required steps to establish whether therapeutic ultrasound can serve as the basis for a new, long term, reversible male contraceptive.

## Competing interests

The authors declare that they have no competing interests.

## Authors' contributions

JKT directed research for Study 2, treated animals, analyzed histology, analyzed sperm parameters, and wrote the manuscript; PAD, CMG, RCG, and TSG designed, performed or analyzed in vitro ultrasound experiments, MGO, PAD, GJM and DCS analyzed data and directed Pilot Studies and Study 1; MAS treated animals, performed necropsies and supervised all animal experiments performed at ILS; EJRS and KGH treated animals, performed necropsies and analyzed sperm. All authors participated in experimental design and read and approved the final manuscript. The authors declare that no actual or potential conflict of interest exists that could inappropriately influence, or be perceived to influence, this work. All authors read and approved the final manuscript.

## Supplementary Material

Additional file 1 **Figure S1**: Beam field maps of the 5 cm^2 ^and 10 cm^2 ^transducers. The ME7413 (5 cm^2^) and ME7410 (10 cm^2^) transducers were mapped at 1 MHz frequency and 1 W/cm^2 ^power at a variety of distances from the transducer face. Beam field maps were identical regardless of the coupling medium used (DW, degassed DW or 3% saline). Click here for file

Additional file 2 **Figure S2**: Two treatments with 3 MHz ultrasound uniformly depleted the testis of spermatocytes & spermatids. This is the same testis depicted in Figure 5. Two consecutive fifteen minute treatments of 3 MHz ultrasound at 2.2 W/cm^2 ^were applied through degassed, distilled water held at 37°C. This magnification emphasizes the uniformity of the ultrasound effect. Only 5% of the seminiferous tubules were observed to have thermal damage while the remaining tubules were depleted of spermatocytes and spermatids. This treatment would have provided at least two months of infertility since spermatogonia require that much time to enter the epididymis as sperm. Click here for file
