# Supplementary material for: Therapeutic ultrasound as a potential male contraceptive: power, frequency and temperature required to deplete rat testes of meiotic cells and epididymides of sperm determined using a commercially available system
Source: Reprod Biol Endocrinol. 2012 Jan 30;10:7. doi: 10.1186/1477-7827-10-7 (PMC3340307; doi:10.1186/1477-7827-10-7)

Beam scans for sonicator probes

All scans were taken at 1MHz and 1 W/cm^2

Output measurements are in kiloPascals

Results summary

• Both transducers have a relatively “unfocused” beam pattern at the target depth– the -6dB beam width is approximately 1.5-3 cm for each transducer, with the ME7410 (10 sq cm) in general larger than the 7413 (5 sq cm).

• Acoustic pressure output varies with distance by approximately 15% - with the peak pressure (averaged over a 1cm square center region) occurring at 2cm for the 7413 and 3.5 cm (or beyond) for the 7410.

• Pressure output of ~ 300 kPa is a fairly low output pressure – correlating to a mechanical index of only 0.3 – below what is usually considered the threshold for bubble cavitation and bio-effects (greater than 0.7).

• Because of the general response of the transducer in the near field – it would be recommended to move the transducer or sample during treatment for even exposure.

• Even though the transducer elements are larger – ultrasound emission is concentrated only in the center region (roughly 1.5-3 cm, depending on distance) from the transducer face, so sample alignment is important.

• A small region (~1mm) near the center of the beam in some locations had a pressure that was 50% greater than the surrounding area. As this could confound results, it would be recommended performing testing where this peak is not present (1 or 3 cm for the 7413 or 2 or 3.5 cm for the 7410).

ME7413 5mm away from transducer

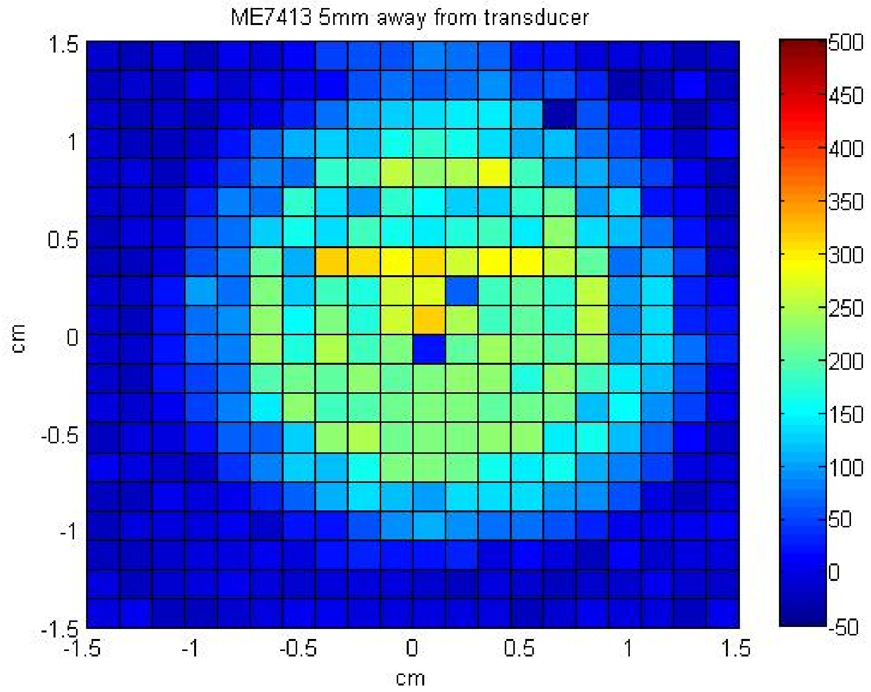

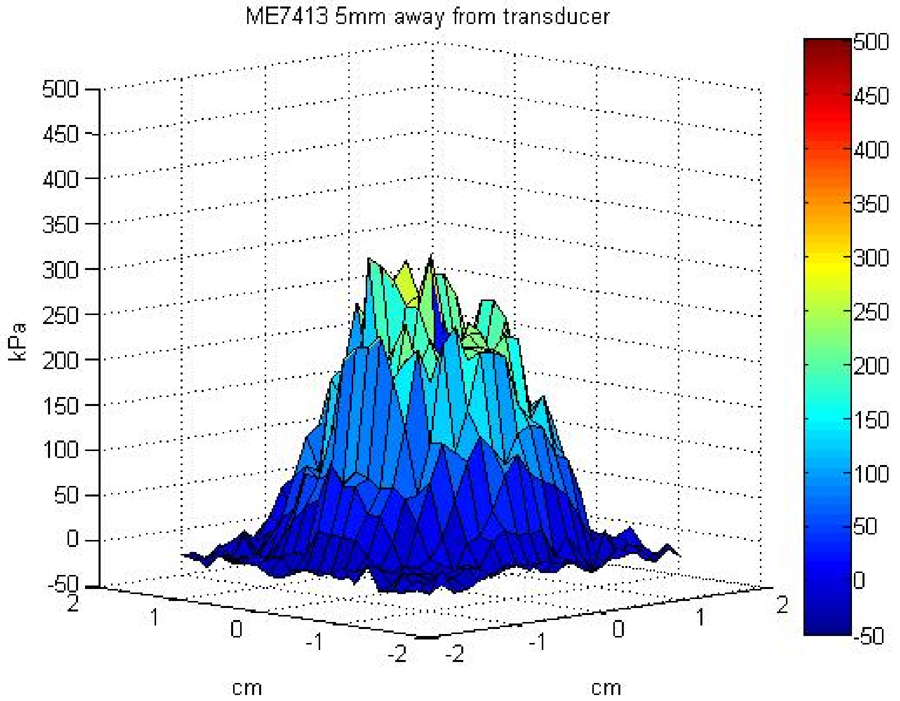

-6 dB beamwidth: 18 mm

ME7413 10mm away from transducer

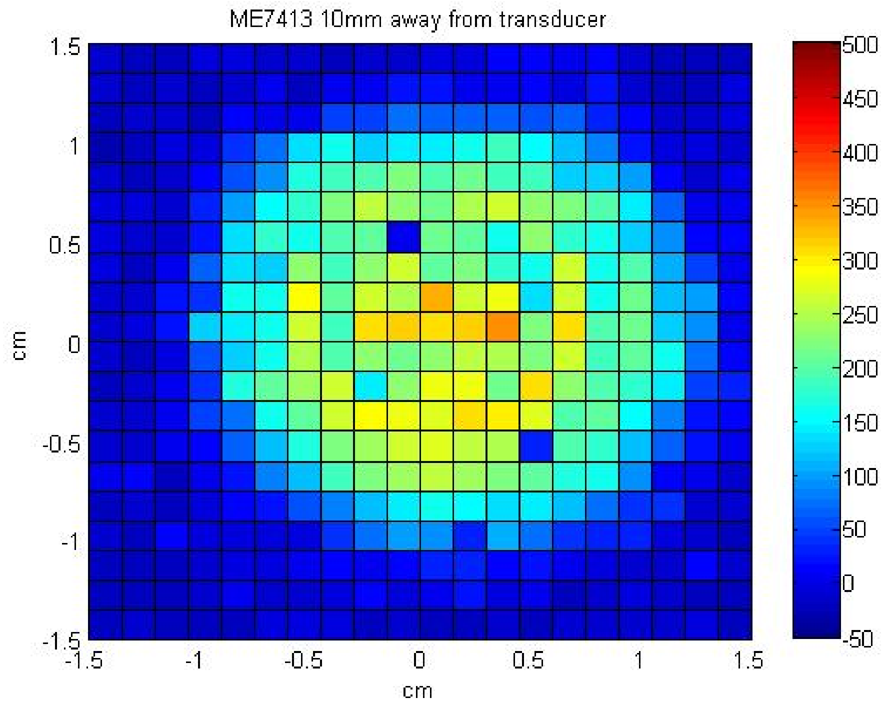

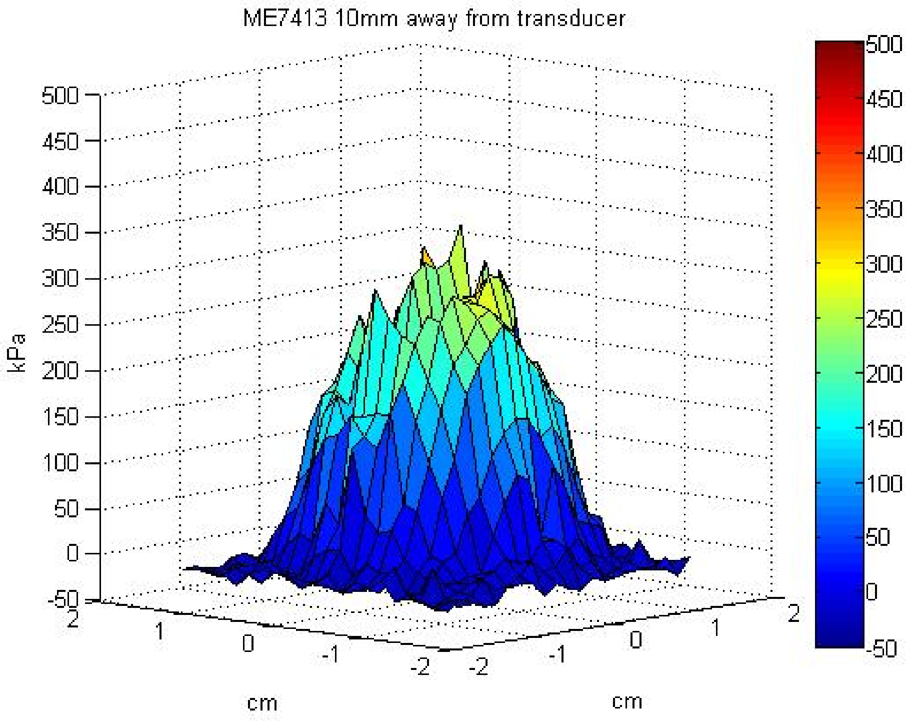

-6 dB beamwidth: 19.5 mm

ME7413 15mm away from transducer

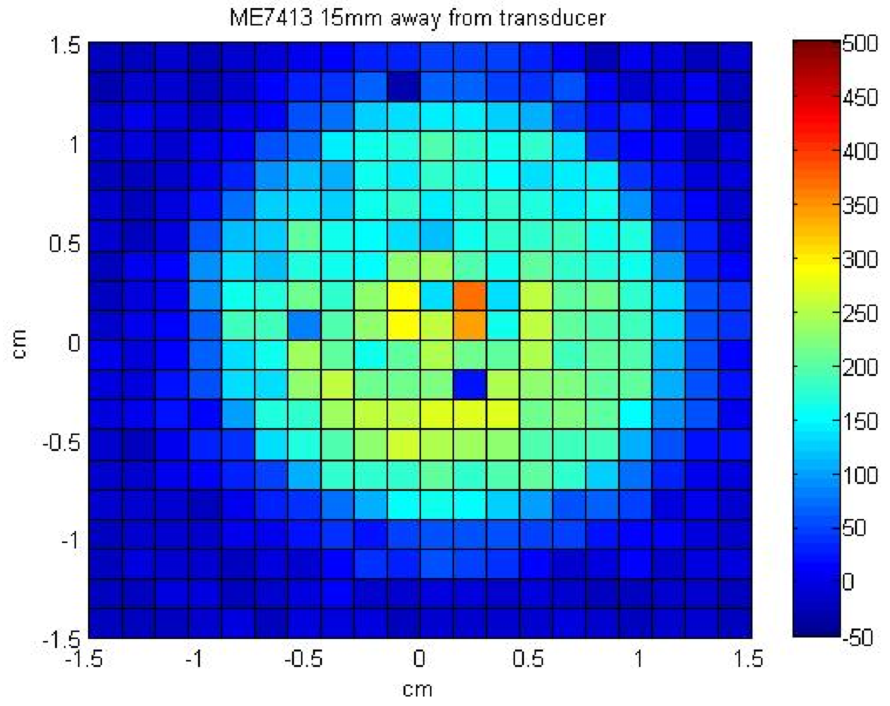

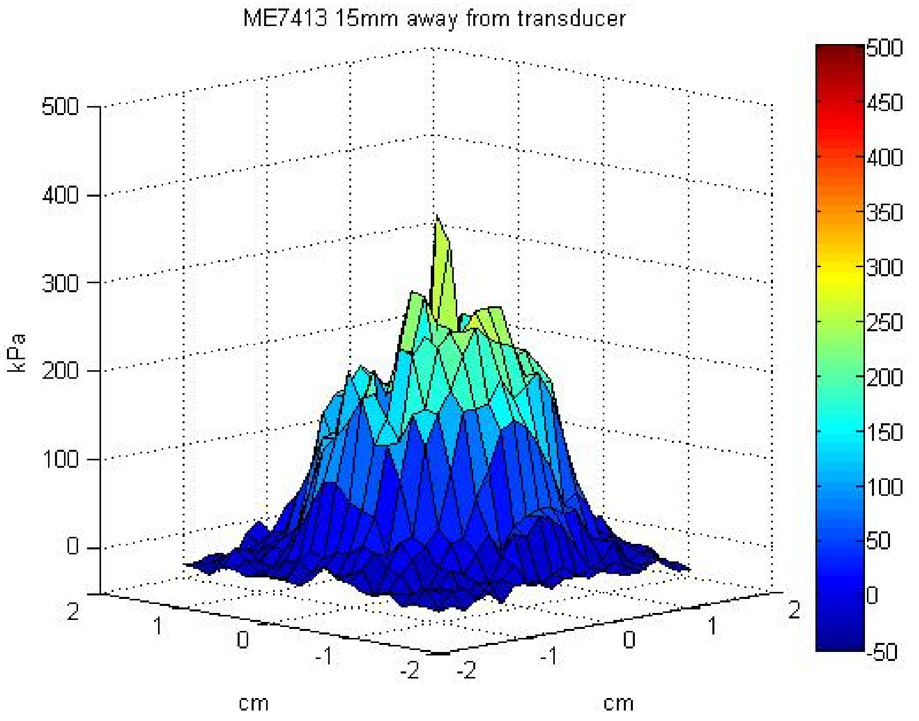

-6 dB beamwidth: 16.5 mm

ME7413 20mm away from transducer

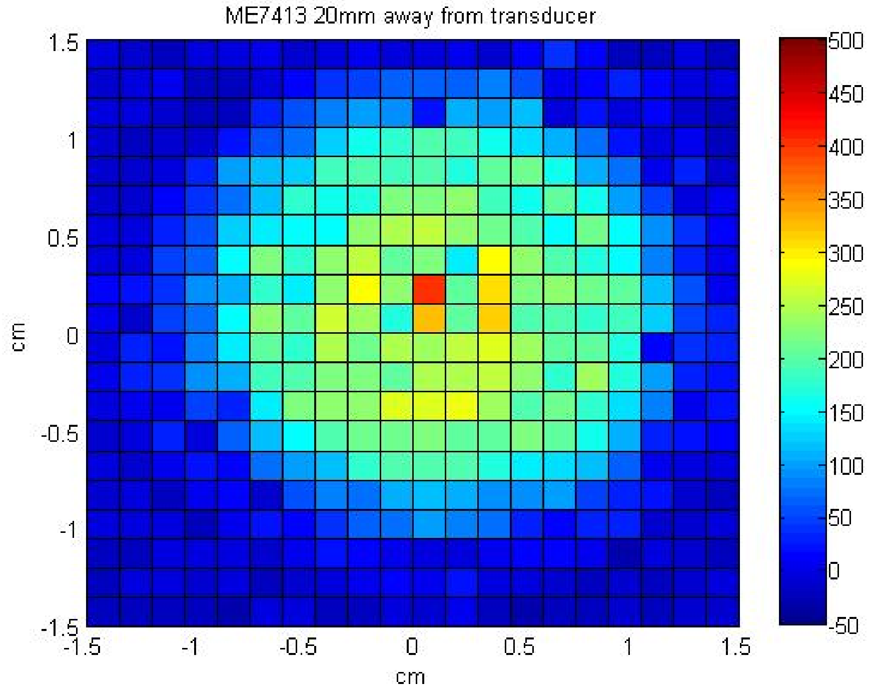

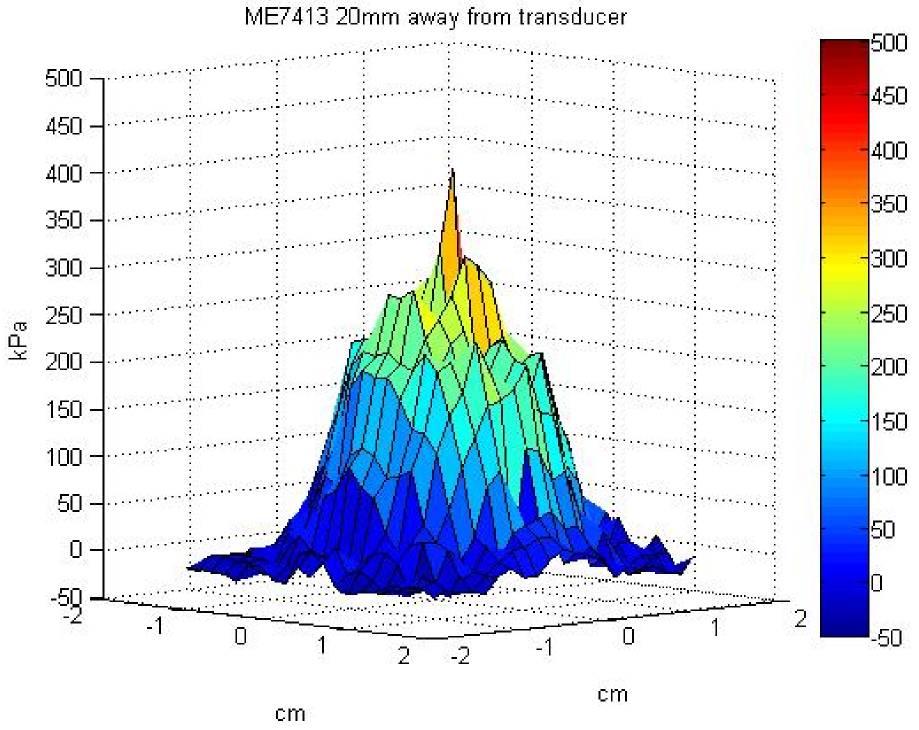

-6 dB beamwidth: 16.5 mm

ME7413 25mm away from transducer

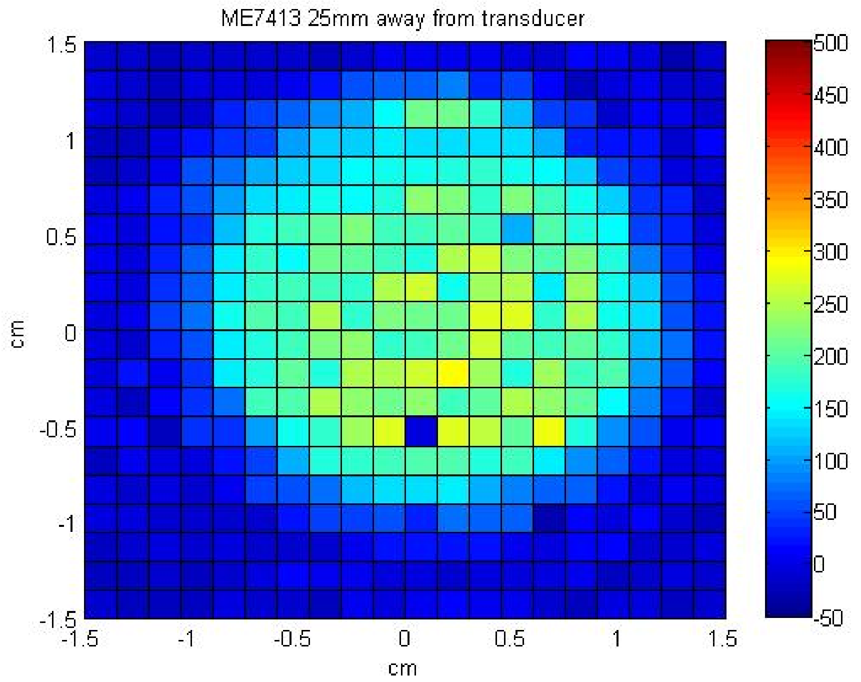

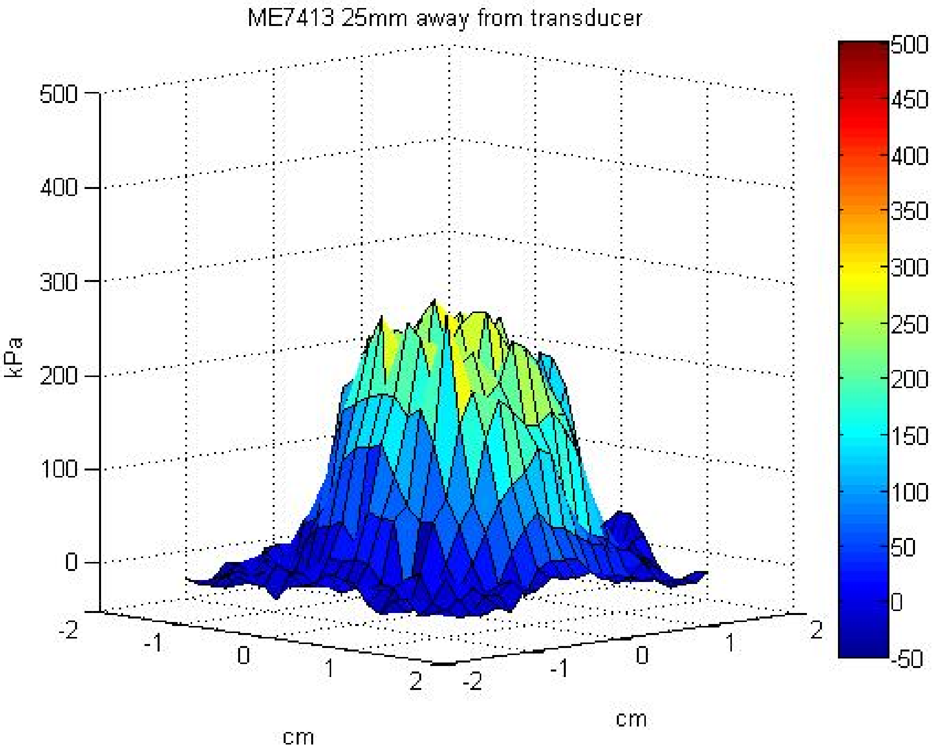

-6 dB beamwidth: 19.5 mm

ME7413 30mm away from transducer

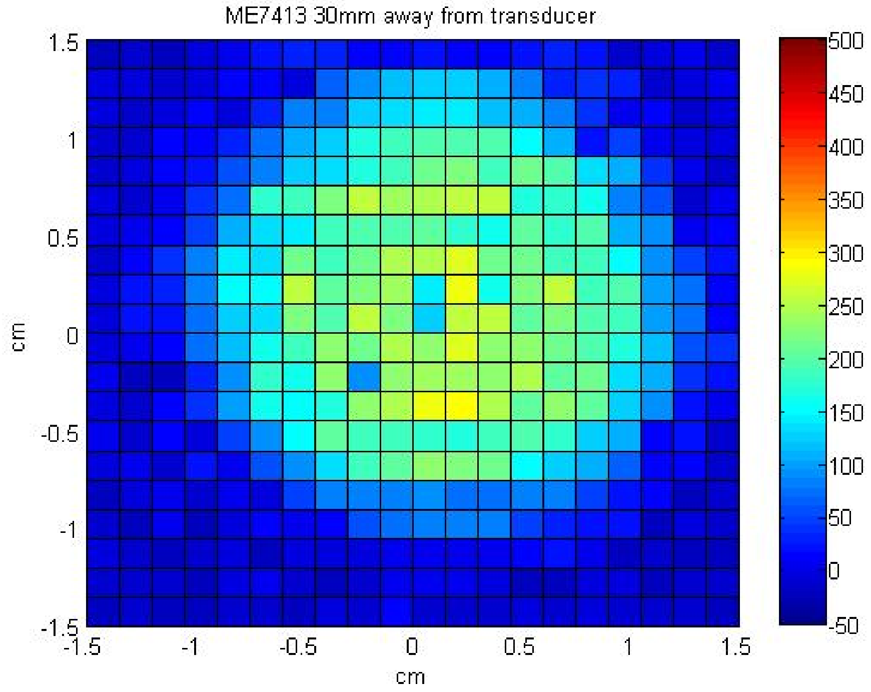

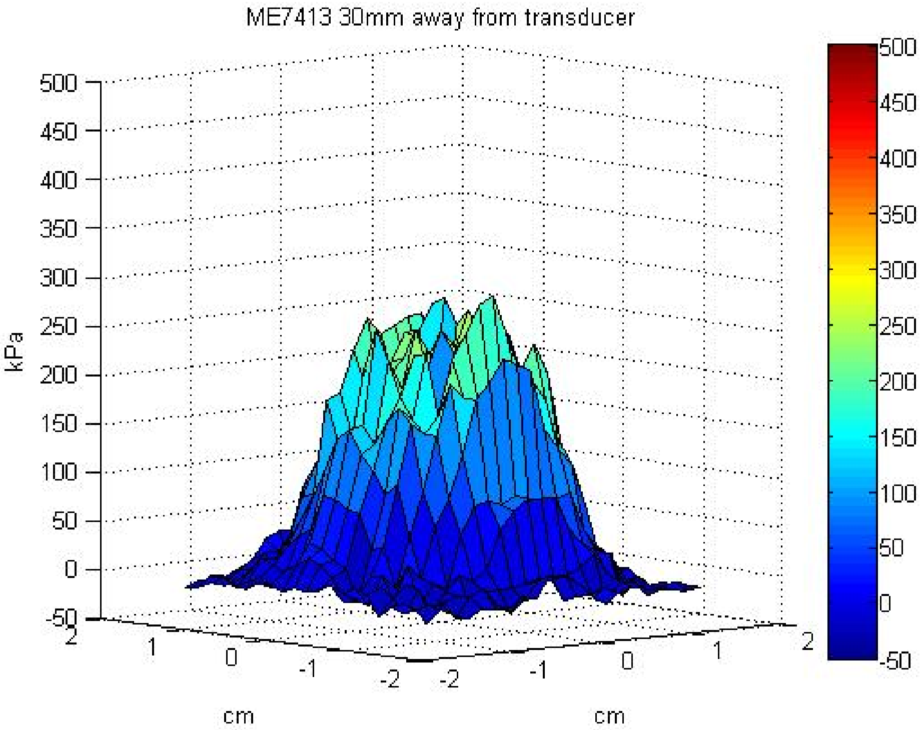

-6 dB beamwidth: 19.5 mm

ME7413 35mm away from transducer

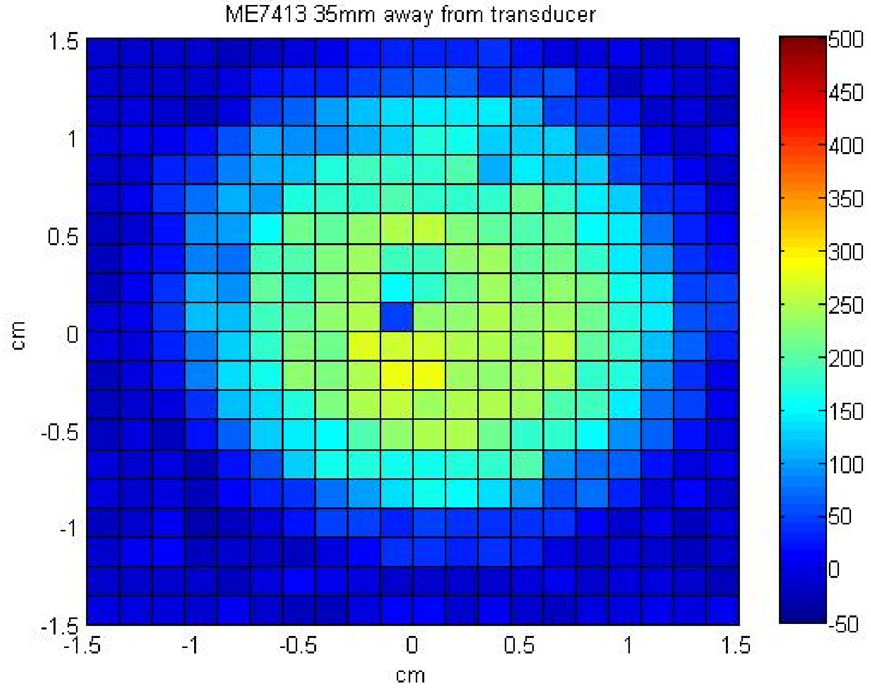

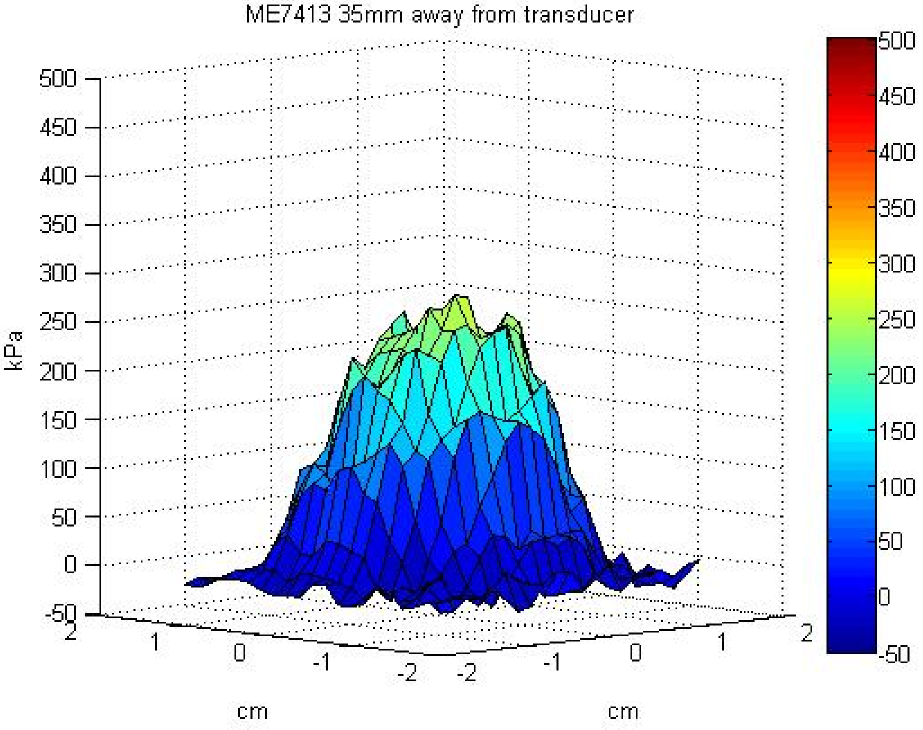

-6 dB beamwidth: 19.5 mm

ME7413 maximum pressure

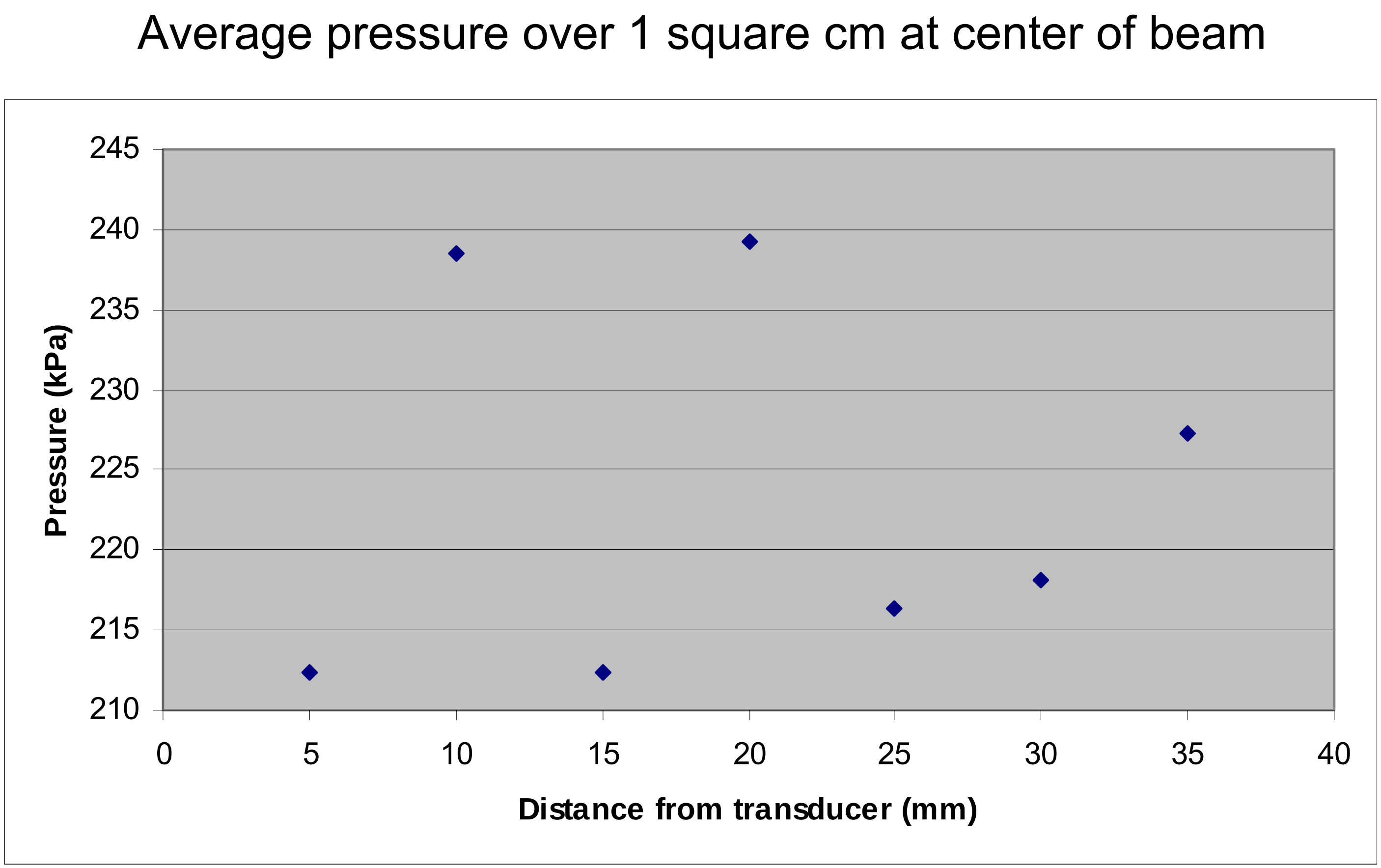

ME7410 5mm away from transducer

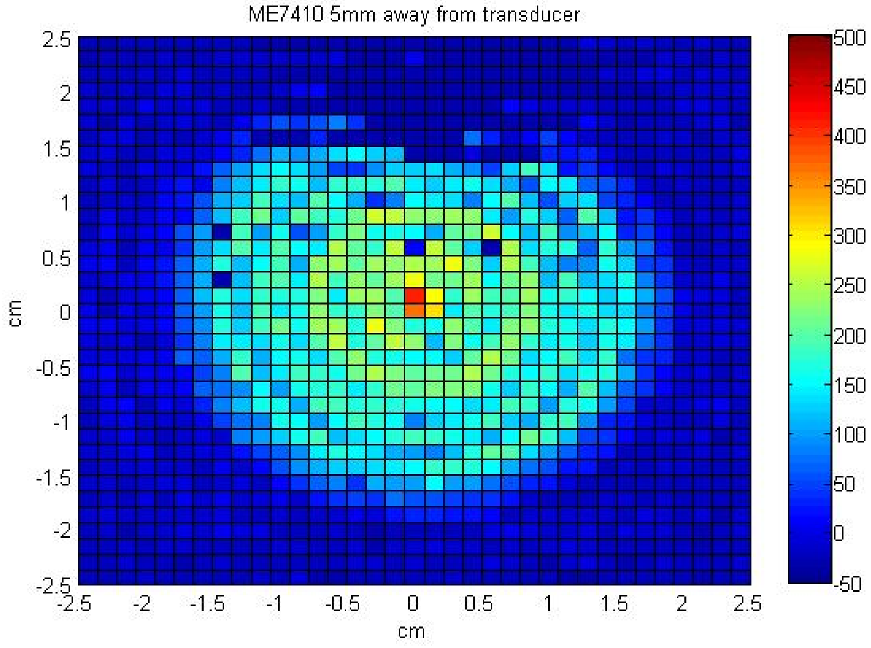

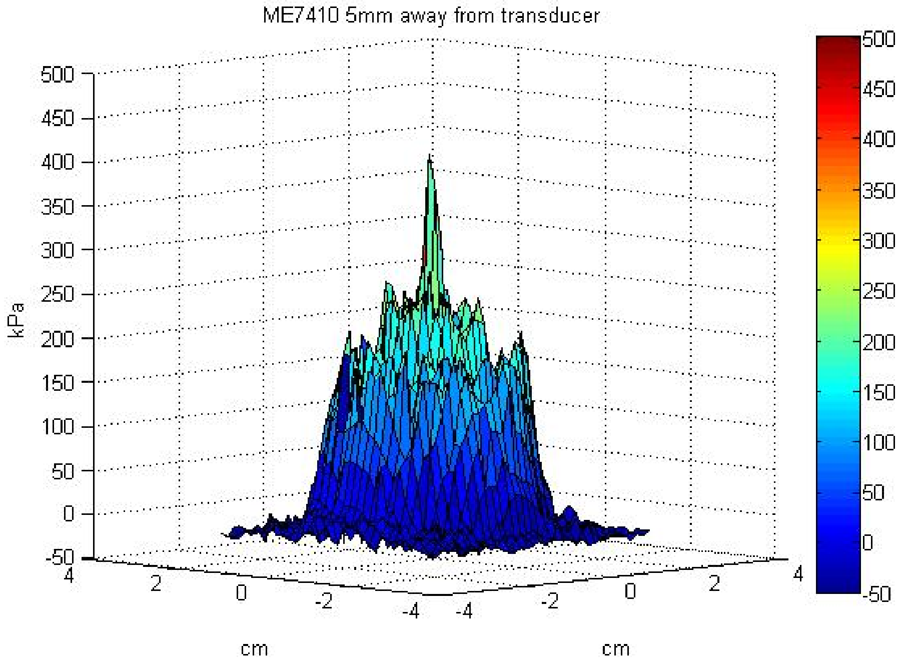

-6 dB beamwidth: 21.4 mm

ME7410 10mm away from transducer

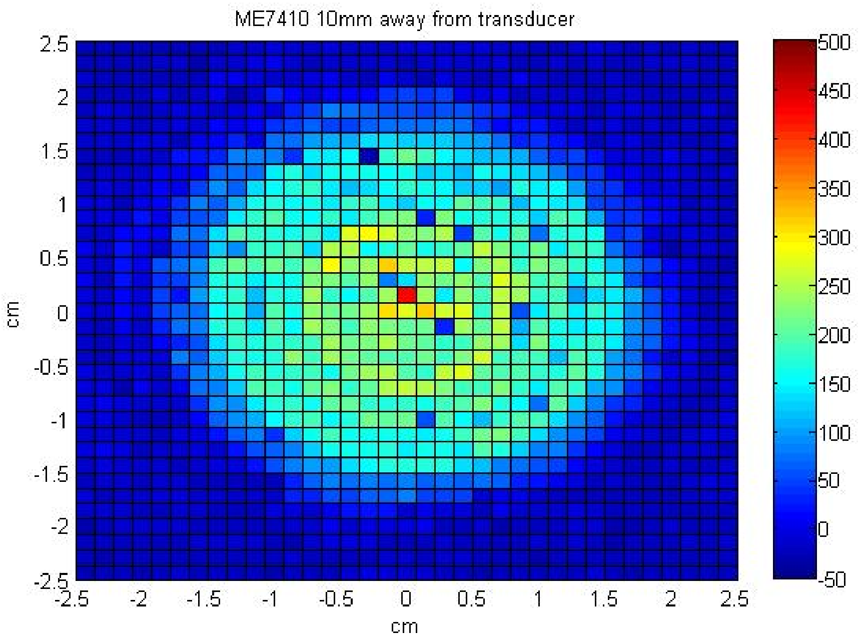

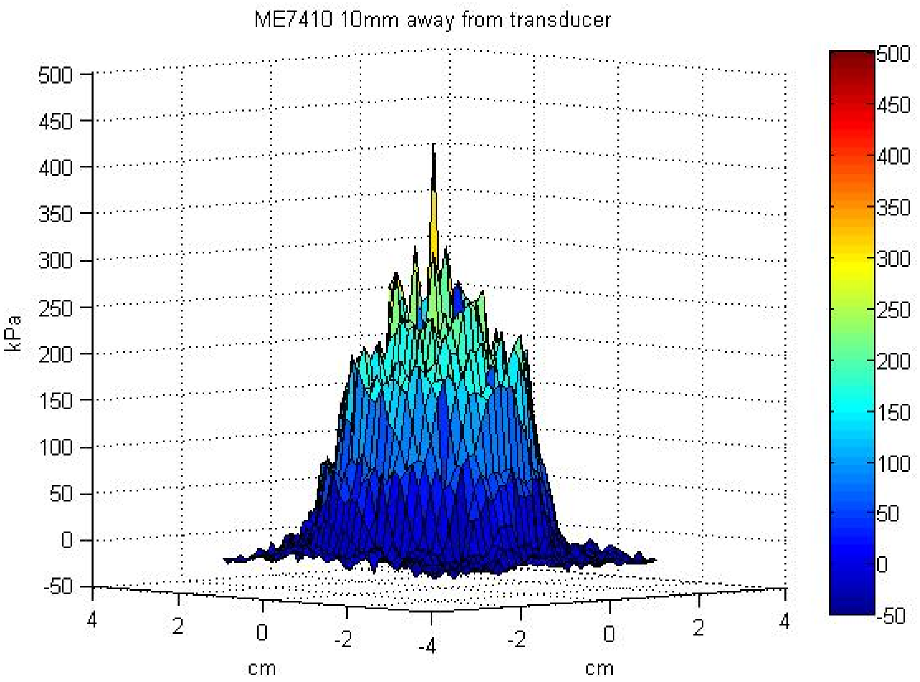

-6 dB beamwidth: 21.4 mm

ME7410 15mm away from transducer

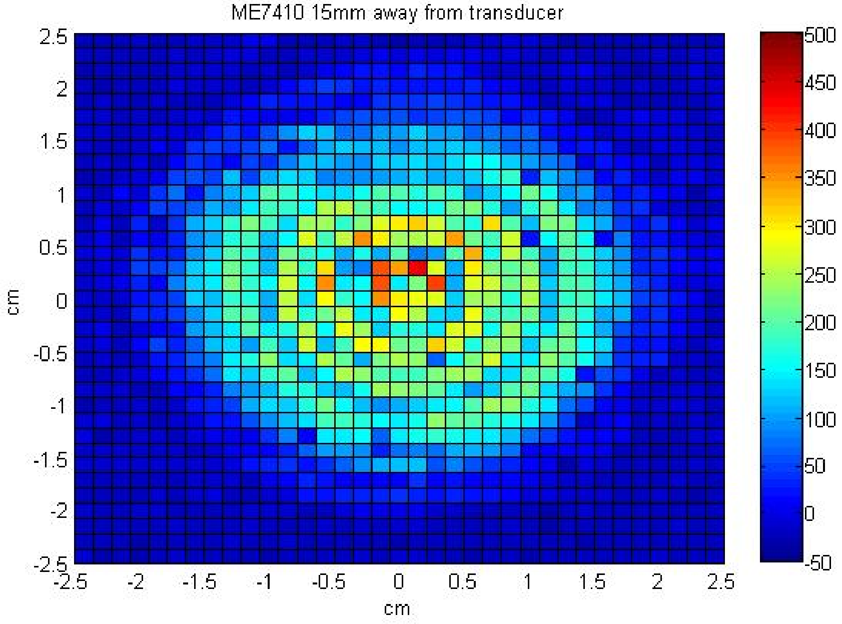

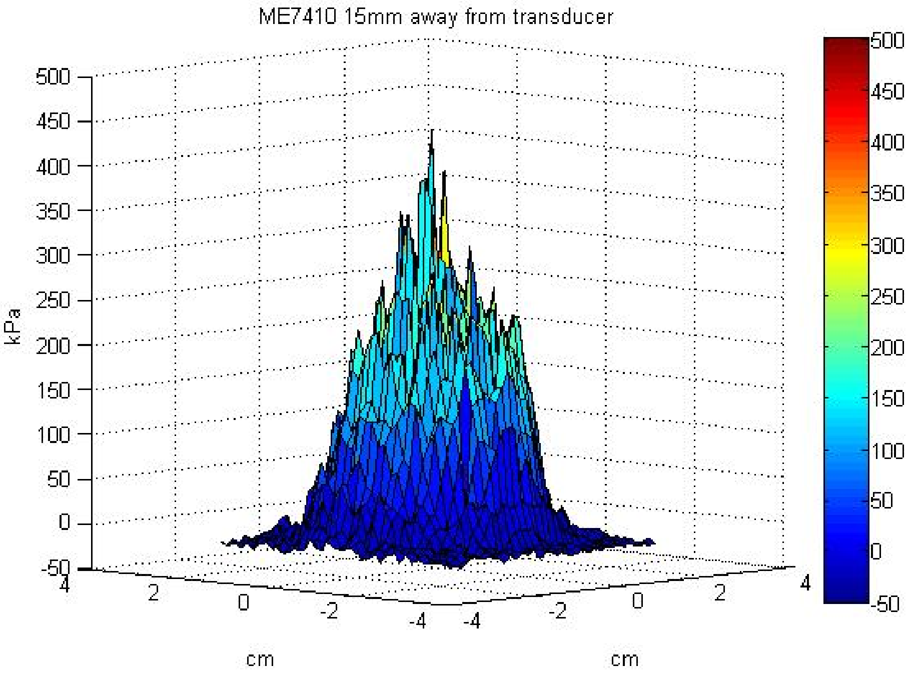

-6 dB beamwidth: 24.3 mm

ME7410 20mm away from transducer

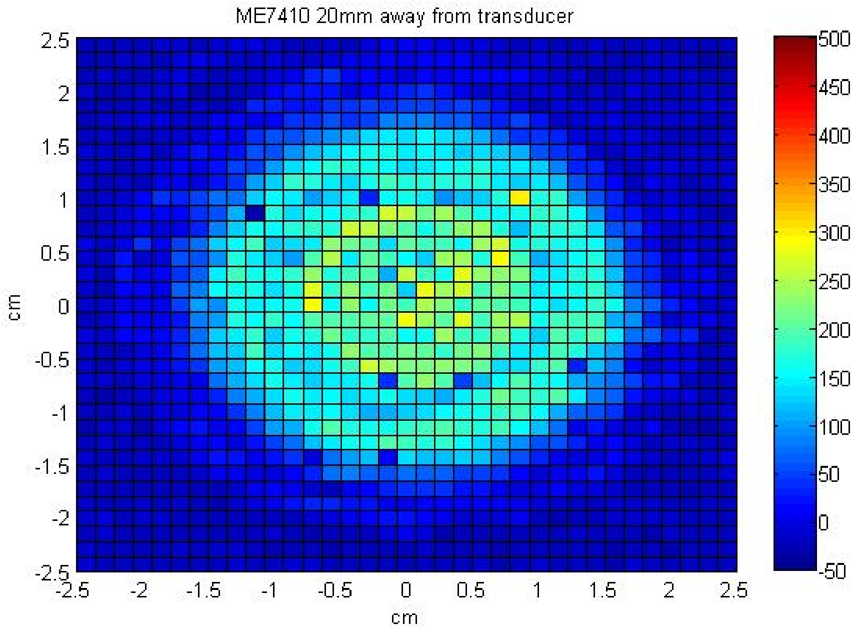

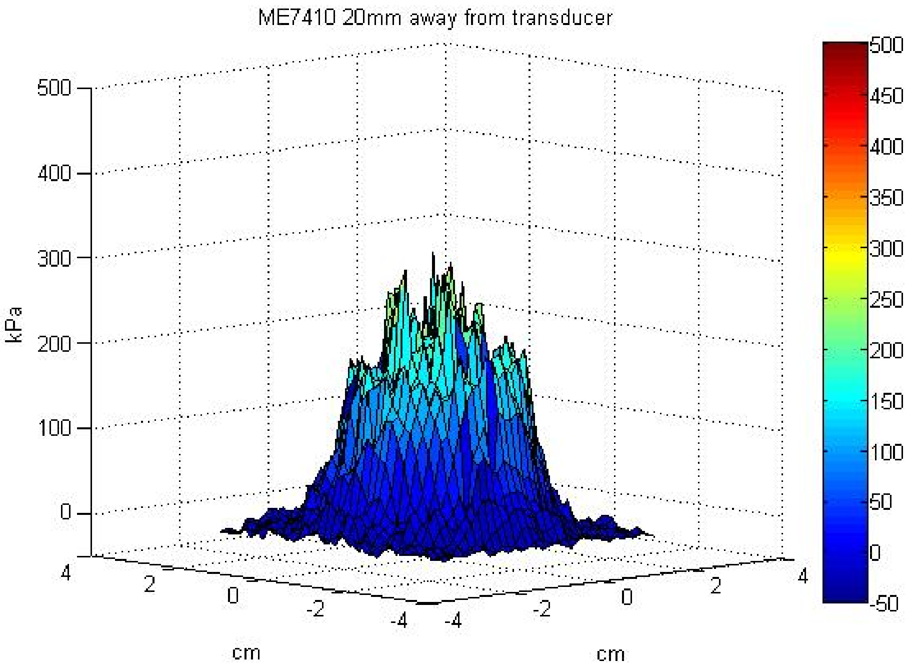

-6 dB beamwidth: 30 mm

ME7410 25mm away from transducer

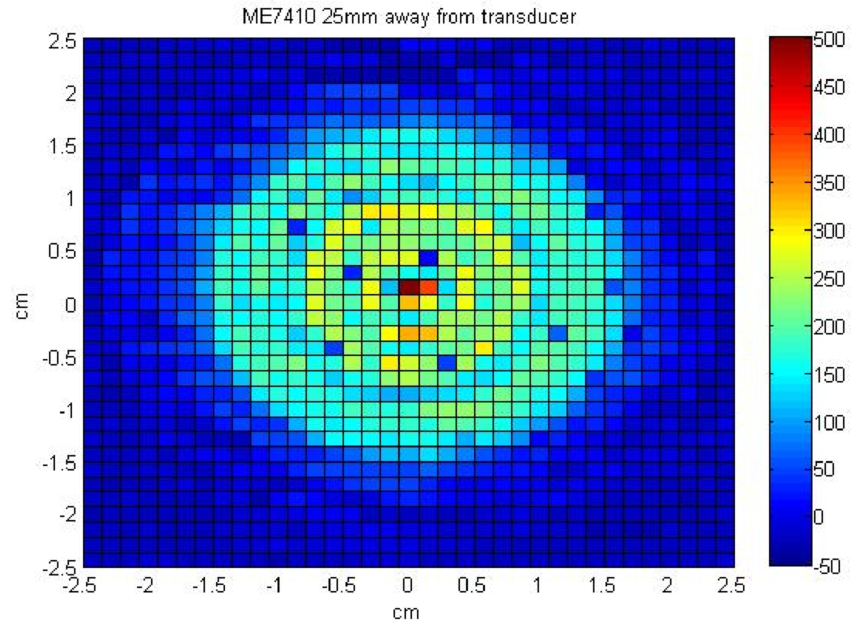

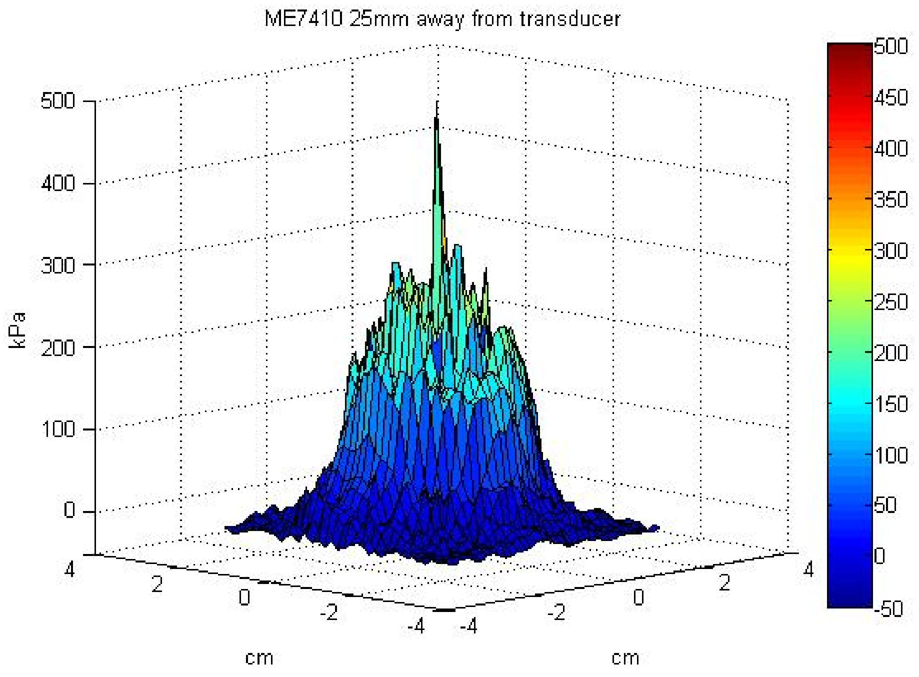

-6 dB beamwidth: 17.1 mm

ME7410 30mm away from transducer

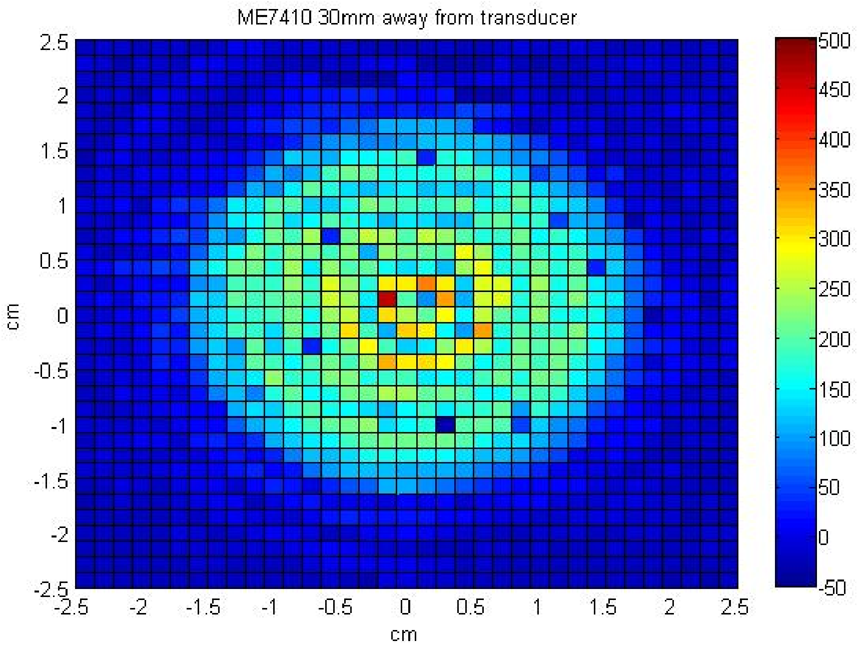

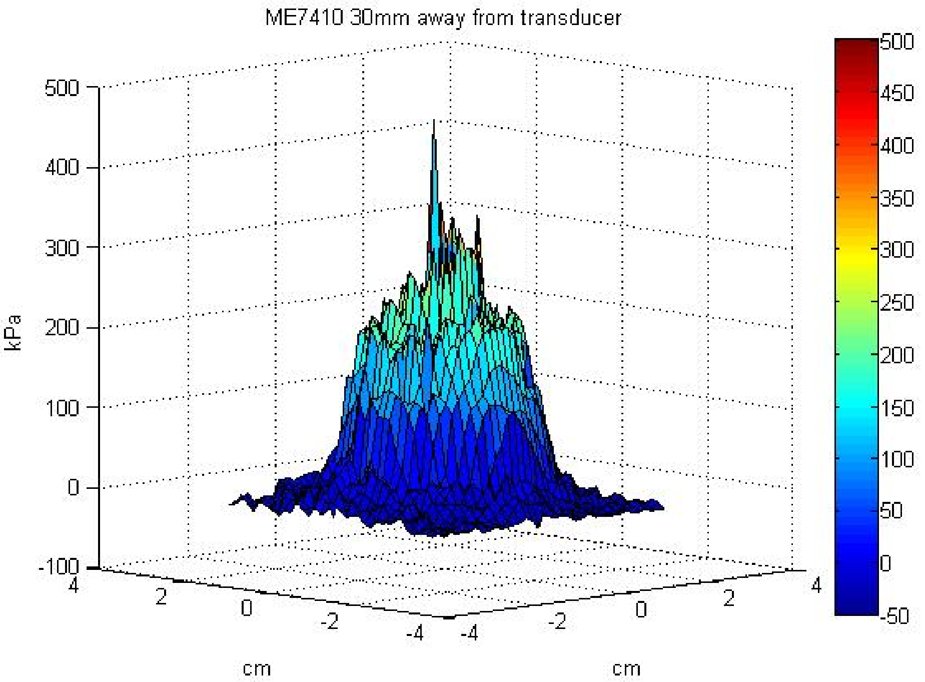

-6 dB beamwidth: 20 mm

ME7410 35mm away from transducer

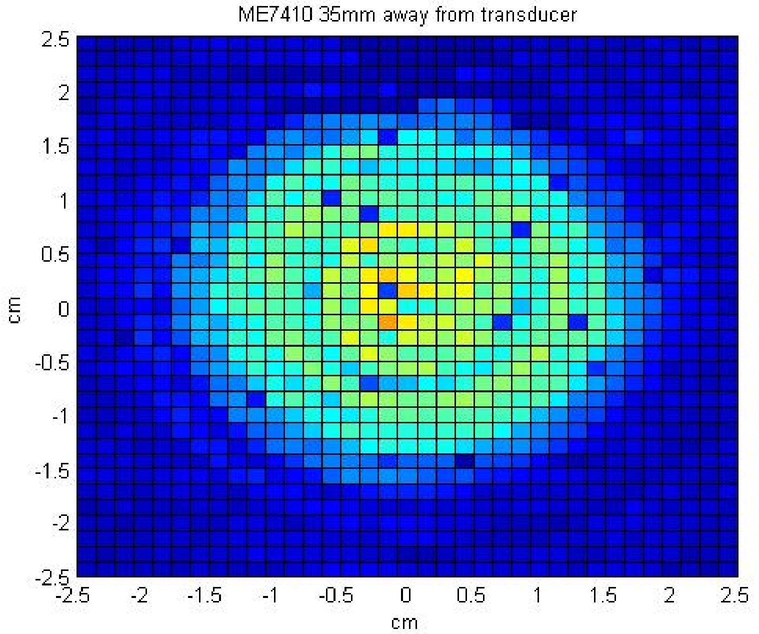

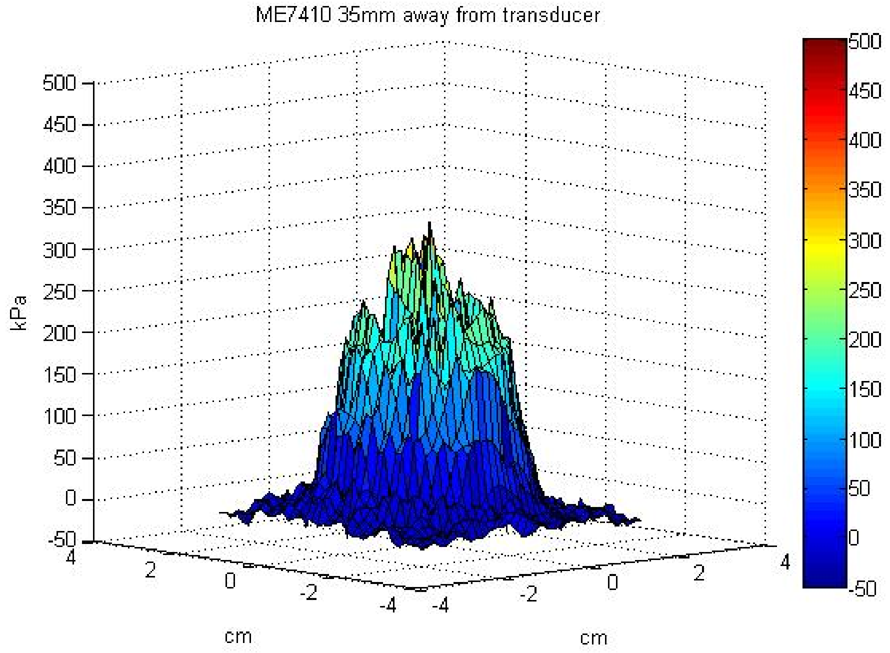

-6 dB beamwidth: 28.6 mm

ME7410 maximum pressure

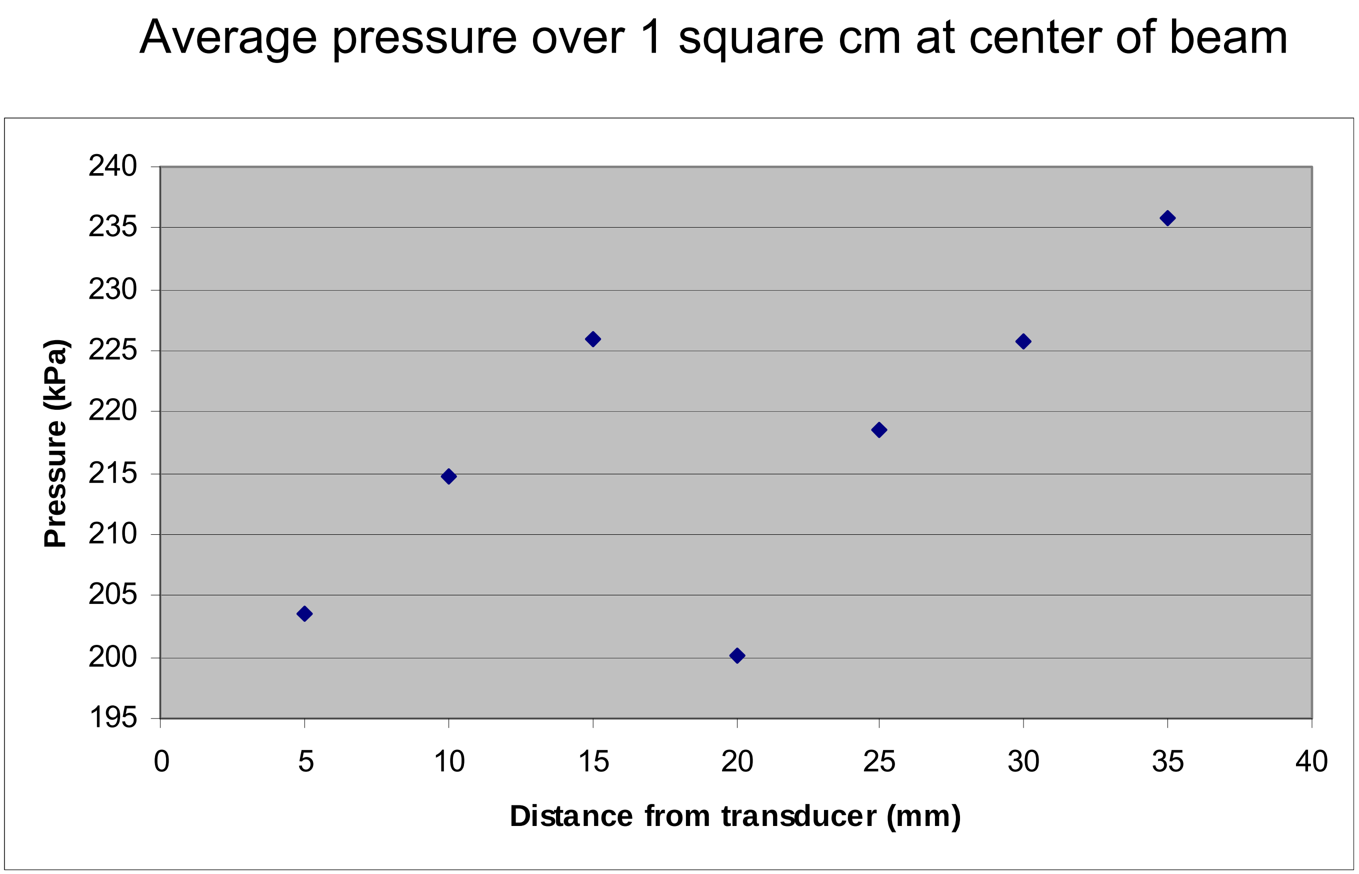

Supplement: Additional file 1 — Figure S1: Beam field maps of the 5 cm2 and 10 cm2 transducers. The ME7413 (5 cm2) and ME7410 (10 cm2) transducers were mapped at 1 MHz frequency and 1 W/cm2 power at a variety of distances from the transducer face. Beam field maps were identical regardless of the coupling medium used (DW, degassed DW or 3% saline). [file 1477-7827-10-7-S1.PDF]
