# Supplementary figures and images for: Therapeutic ultrasound as a potential male contraceptive: power, frequency and temperature required to deplete rat testes of meiotic cells and epididymides of sperm determined using a commercially available system
Source: Reprod Biol Endocrinol. 2012 Jan 30;10:7. doi: 10.1186/1477-7827-10-7 (PMC3340307; doi:10.1186/1477-7827-10-7)

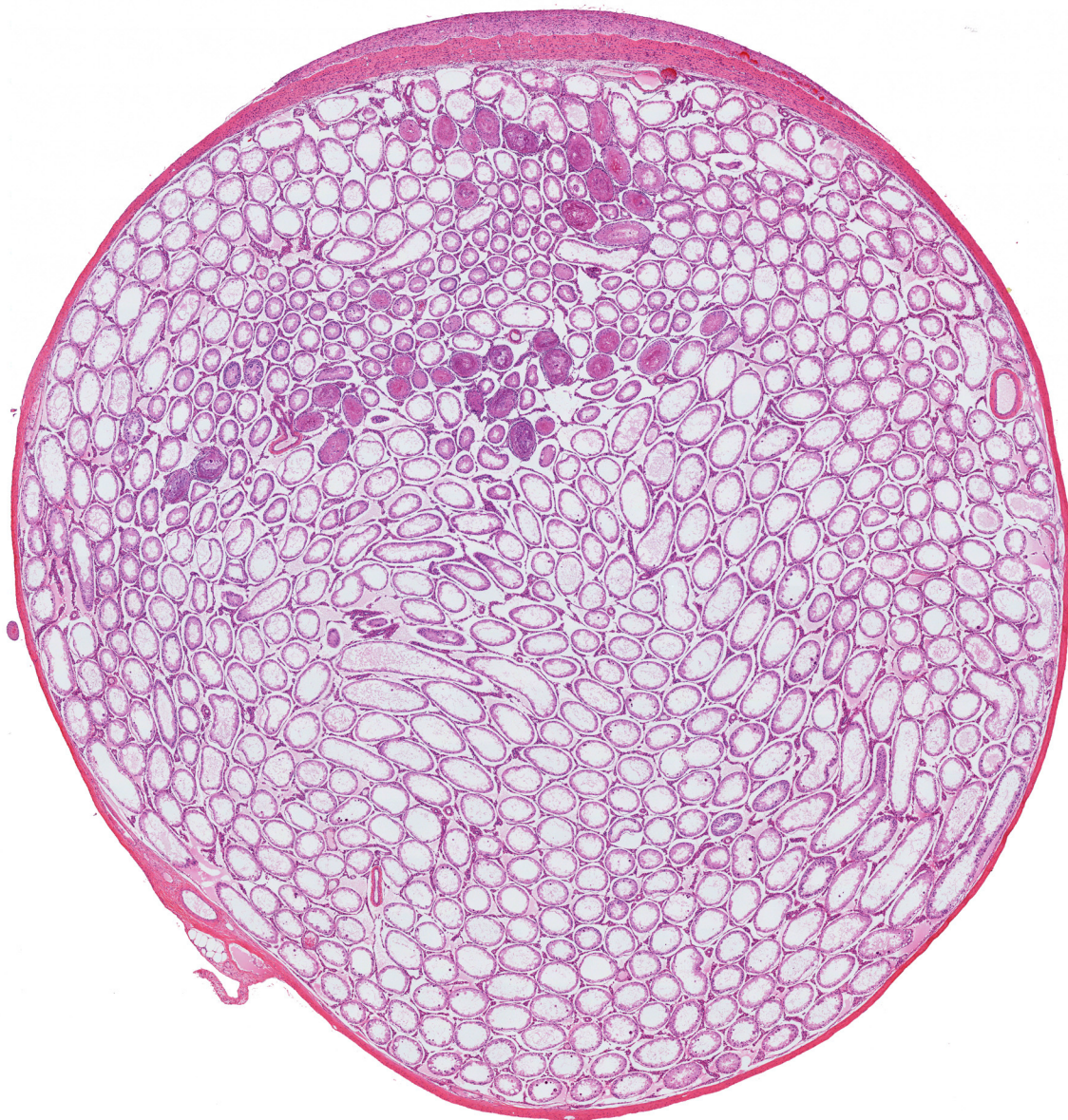

Supplement: Additional file 2 — Figure S2: Two treatments with 3 MHz ultrasound uniformly depleted the testis of spermatocytes & spermatids. This is the same testis depicted in Figure 5. Two consecutive fifteen minute treatments of 3 MHz ultrasound at 2.2 W/cm2 were applied through degassed, distilled water held at 37°C. This magnification emphasizes the uniformity of the ultrasound effect. Only 5% of the seminiferous tubules were observed to have thermal damage while the remaining tubules were depleted of spermatocytes and spermatids. This treatment would have provided at least two months of infertility since spermatogonia require that much time to enter the epididymis as sperm. [file 1477-7827-10-7-S2.PDF]
